# YAP1 preserves tubular mitochondrial quality control to mitigate diabetic kidney disease

**DOI:** 10.1016/j.redox.2024.103435

**Published:** 2024-11-23

**Authors:** Siyang Ye, Meng Zhang, Xunhua Zheng, Suchun Li, Yuting Fan, Yiqin Wang, Huajing Peng, Sixiu Chen, Jiayi Yang, Li Tan, Manhuai Zhang, Peichen Xie, Xiaoyan Li, Ning Luo, Zhipeng Wang, Leigang Jin, Xiaoping Wu, Yong Pan, Jinjin Fan, Yi Zhou, Sydney C.W. Tang, Bin Li, Wei Chen

**Affiliations:** a Department of Nephrology, The First Affiliated Hospital, Sun Yat-sen University, Guangzhou, 510080, China; b NHC Key Laboratory of Clinical Nephrology (Sun Yat-sen University) and Guangdong Provincial Key Laboratory of Nephrology, Guangzhou, 510080, China; c State Key Laboratory of Pharmaceutical Biotechnology, LKS Faculty of Medicine, The University of Hong Kong, Hong Kong, China; d Department of Pharmacology and Pharmacy, LKS Faculty of Medicine, The University of Hong Kong, Hong Kong, China; e Department of Medicine, LKS Faculty of Medicine, The University of Hong Kong, Hong Kong, China; f Department of Pathophysiology, School of Basic Medical Science, Shenzhen University Medical School, Shenzhen, 518000, China; g Division of Nephrology, Department of Medicine, LKS Faculty of Medicine, The University of Hong Kong, Hong Kong, China

**Keywords:** Diabetic kidney disease, Hippo signaling pathway, Yes-associated protein 1, Mitochondria, Chemokine, Macrophage

## Abstract

Renal tubule cells act as a primary site of injury in diabetic kidney disease (DKD), with dysfunctional mitochondrial quality control (MQC) closely associated with progressive kidney dysfunction in this context. Our investigation delves into the observed inactivation of yes-associated protein 1 (YAP1) and consequential dysregulation of MQC within renal tubule cells among DKD subjects through bioinformatic analysis of transcriptomics data from the Gene Expression Omnibus (GEO) dataset. Receiver operating characteristic curve analysis unequivocally underscores the robust diagnostic accuracy of YAP1 and MQC-related genes for DKD. Furthermore, we observed YAP1 inactivation, accompanied by perturbed MQC, within cultured tubule cells exposed to high glucose (HG) and palmitic acid (PA). This pattern was also evident in the tubulointerstitial compartment of kidney sections from biopsy-approved DKD patients. Additionally, renal tubule cell-specific *Yap1* deletion exacerbated kidney injury in diabetic mice. Mechanistically, *Yap1* deletion disrupted MQC, leading to mitochondrial aberrations in mitobiogenesis and mitophagy within tubule cells, ultimately culminating in histologic tubular injury. Notably, *Yap1* deletion-induced renal tubule injury promoted the secretion of C-X-C motif chemokine ligand 1 (CXCL1), potentially augmenting M1 macrophage infiltration within the renal microenvironment. These multifaceted events were significantly ameliorated by administrating the YAP1 activator XMU-MP-1 in DKD mice. Consistently, bioinformatic analysis of transcriptomics data from the GEO dataset revealed a noteworthy upregulation of tubule cells-derived chemokine CXCL1 associated with macrophage infiltration among DKD patients. Crucially, overexpression of YAP1 via adenovirus transfection sustained mitochondrial membrane potential, mtDNA copy number, oxygen consumption rate, and activity of mitochondrial respiratory chain complex, but attenuated mitochondrial ROS production, thereby maintaining MQC and subsequently suppressing CXCL1 generation within cultured tubule cells exposed to HG and PA. Collectively, our study establishes a pivotal role of tubule YAP1 inactivation-mediated MQC dysfunction in driving DKD progression, at least in part, facilitated by promoting M1 macrophage polarization through a paracrine-dependent mechanism.

## Highlights

• A robust diagnostic model incorporating YAP1 and mitochondrial quality control (MQC)-related genes demonstrates high accuracy in detecting DKD.
• In DKD, YAP1 inactivation correlates with impaired MQC in renal tubule cells.
• Deletion of *Yap1* in tubule cells disrupts MQC, leading to increased secretion of CXCL1 and ultimately enhanced macrophage infiltration.
• YAP1 activation preserves MQC homeostasis, subsequently reducing renal injury and M1 macrophage infiltration.
• Targeting the Hippo signaling pathway shows promise in DKD prevention and treatment by addressing YAP1 inactivation-mediated MQC dysfunction in renal tubule cells.

## Graphical abstract

**Figure:**
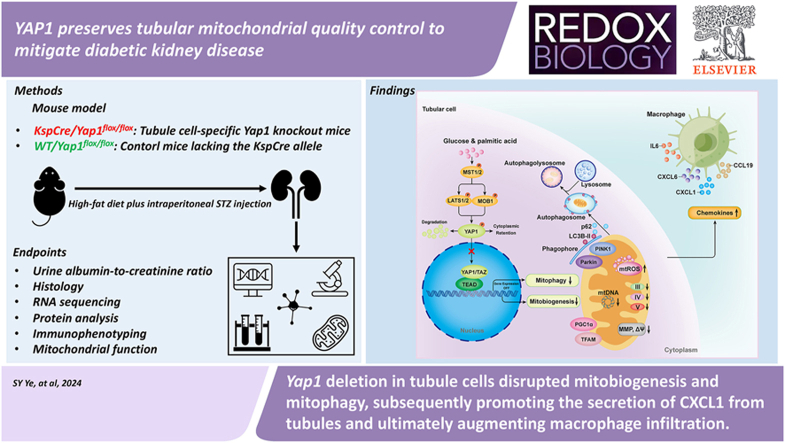

## 1 Introduction

Diabetic kidney disease (DKD) is a prominent long-term microvascular complication that afflicts individuals with both type 1 and type 2 diabetes mellitus. Despite the application of current best clinical management strategies, which encompass renin-angiotensin system blockade, and stringent control of both hypertension and hyperglycemia, DKD persists as a primary precipitator of end-stage renal disease (ESRD) necessitating renal replacement therapy [1]. The limited efficacy of existing therapeutic modalities underscores the presence of a substantial treatment gap, emphasizing the imperative need for a more comprehensive understanding of the pathophysiological events and molecular mechanisms underpinning the progression of DKD. Recent investigations have introduced the concept of “diabetic tubulopathy”, which spotlights the pivotal role of tubular dysfunction in the genesis and progression of DKD [[1], [2], [3]]. According to this tubulocentric perspective on DKD, hyperglycemia exposes tubular structures to augmented glucose loads and reabsorption, resulting in glomerular hyperfiltration via the tubuloglomerular feedback system [4,5]. This process is intricately linked to inflammation or fibrosis and can incite renal oxidative stress, cortical interstitial inflammation, hypoxia, and tubulointerstitial fibrosis, thereby propelling the inexorable advancement of renal pathology [2,4,[6], [7], [8]].

Renal tubule cells demand a substantial number of mitochondria to fulfill their high-energy requirements and sustain aerobic metabolism through oxidative phosphorylation [3,7,9]. The preservation of mitochondrial integrity, encompassing quality, quantity, and functionality, hinges on the efficient operation of mitochondrial quality control (MQC) mechanisms [[10], [11], [12]]. These mechanisms operate at both the molecular and organelle levels and encompass mitochondrial protein quality control, the regulation of mitochondrial proteases and molecular chaperones, mitochondrial dynamics, mitophagy, and mitobiogenesis [8,11,13]. Given the intimate association between mitochondria and tubule cells, mitochondrial dysfunction has emerged as a pivotal factor in the onset and progression of diabetic tubulopathy [3,8,12,14,15]. However, the specific MQC mechanisms involved in diabetic tubulopathy cells remain largely uncharted territory.

Recent research has illuminated the contribution of overabundant proinflammatory M1 macrophages and insufficient anti-inflammatory M2 macrophages to the development of inflammation and fibrosis in DKD [[16], [17], [18]]. In response to diabetic stress, tubule cells acquire a secretory phenotype and secrete various chemokines, which potentiate the recruitment, proliferation, and polarization of macrophages, further exacerbating the pathology of DKD [[19], [20], [21], [22], [23]]. Therefore, a comprehensive perception of the crosstalk between tubule cells and macrophages in DKD is indispensable for deciphering the intricate immune-mediated processes underlying DKD and for the development of efficacious preventive and therapeutic interventions.

Yes-associated protein 1 (YAP1), a core component of the Hippo signaling pathway, participates in a myriad of biological processes encompassing cell death, oxidative stress, and inflammation [24,25]. Recent investigations have unveiled the involvement of YAP1 in kidney tissues, implicating its pivotal role in the pathogenesis of kidney diseases [[26], [27], [28]]. Nevertheless, although prior studies have elucidated the pathological roles of YAP1 in tubular or mesangial injuries, renal fibrosis, and even renal cell carcinoma, the physiological functions of YAP1 within the kidney, particularly in the context of DKD, remain largely undetermined [29]. The primary objective of this study is to investigate the role and mechanism of renal tubule cell YAP1-mediated MQC in the progression of DKD, with particular attention to their influence on local proinflammatory chemokine secretion, intercellular communication between tubules and macrophages, and the ensuing progression of DKD.

## 2 Materials and methods

### 2.1 Generation of tubule cell-specific Yap1 knockout mice

Renal tubule cell-specific *Yap1* knockout mice (TKO, *KspCre*/*Yap1*^*flox/flox*^) and their control counterparts lacking the *KspCre* allele (CTL, *WT/Yap1*^*flox/flox*^) were bred at the Shanghai Model Organisms Center in China. The generation of TKO mice involved the mating of *Yap1*^*flox/flox*^ mice (C57BL/6Smoc-Yap1^tm1(flox)Smoc^, Cat. No. NM-CKO-200174, Shanghai Model Organisms Center, Inc.) with *KspCre* transgenic mice (B6.Cg-Tg(Cdh16-Cre)91Igr/J*,* RRID: IMSR_JAX: 012237, Jackson Laboratory) that expressed Cre recombinase under the control of kidney-specific-cadherin promoter.

### 2.2 High-fat diet (HFD) and streptozotocin (STZ)-induced DKD mice

The mice were categorized into four groups: (1) CTL mice on a normal diet (CTL + Veh + ND) group (n = 6); (2) TKO mice on a normal diet (TKO + Veh + ND) group (n = 6); (3) CTL mice on a high-fat diet (HFD) with streptozotocin (STZ) administration (CTL + STZ + HFD) group (n = 6); and (4) TKO mice on a HFD with STZ administration (TKO + STZ + HFD) group (n = 6). Mice in the ND or HFD groups were fed either a standard chow diet (Research Diets, New Brunswick, NJ, USA) or a HFD (Research Diets) continuously for 22 weeks. After 8 weeks on the HFD, mice in the STZ + HFD groups were intraperitoneally injected with STZ (40 mg/kg; Sigma‐Aldrich, St. Louis, MO, USA) dissolved in ice-cold sodium citrate (pH 4.5; Phygene, Fuzhou, Fujian, China) for five consecutive days, while the Veh + ND groups received the sodium citrate vehicle.

### 2.3 Statistical analysis

The data values reported in this study are presented as mean ± standard error of mean (SEM). Statistical analyses were performed using unpaired two-tailed Student's t-tests or one-way analysis of variance (ANOVA) followed by least significant difference tests. The SPSS software (version 26) was used to analyze the data. The statistical significance was considered when *P* < 0.05.

Additional details for all methods are provided in **Appendix A. Supplementary data**.

## 3 Results

### 3.1 Transcripts of YAP1 and MQC-related mediators were disrupted in renal tubule cells from diabetic kidney

**Fig. 1:**
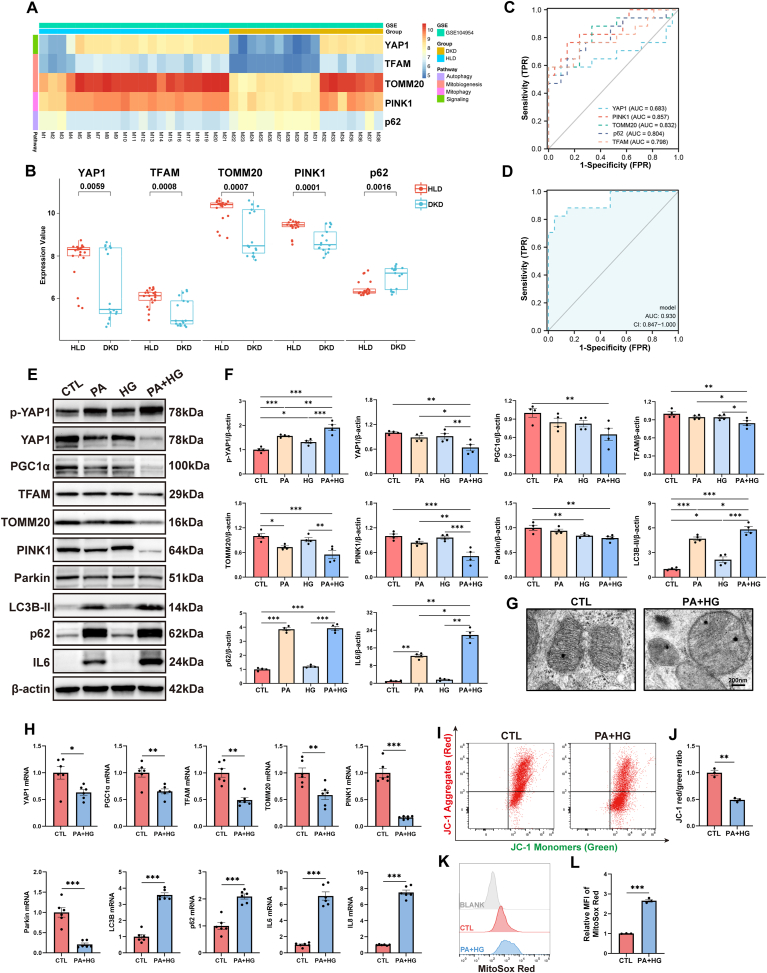
Yes-associated protein 1 (YAP1) and mitochondrial quality control (MQC)-related mediators were disrupted in renal tubule cells from diabetic kidney. (**A, B**) Expression of YAP1 and MQC-related genes in tubulointerstitial compartment of subjects with diabetic kidney disease (DKD) in GSE104954. (**C**) The diagnostic performance of YAP1 and each individual MQC-related genes for DKD. (**D**) The diagnostic performance of a comprehensive predictive model consisted of YAP1 and MQC-related genes for DKD. (**E, F**) Representative Western blots and quantification of p-YAP1, YAP1, MQC-related mediators, and interleukin 6 (IL6) in HK-2 cells exposed to palmitic acid and high glucose (PA + HG). (**G**) Transmission electron microscopy images of mitochondria within HK-2 cell with or without PA + HG treatment. Bar = 200 nm. (**H**) Transcripts of *YAP1*, MQC-related mediators, and proinflammatory genes were measured by quantitative real-time polymerase chain reaction. (**I, J**) Representative plots and quantification of flow cytometry analysis for JC-1 staining in HK-2 cells with or without PA + HG treatment. The JC-1 red/green fluorescence ratio was calculated to represent mitochondrial membrane potential. (**K, L**) Representative plots and quantification of flow cytometry analysis for MitoSox Red staining in HK-2 cells with or without PA + HG treatment. The mean fluorescence intensity of MitoSox Red was calculated to represent mitochondrial reactive oxygen species level. Data are expressed as the mean ± SEM. All experiments were repeated at least three times. ∗P < 0.05; ∗∗P < 0.01; ∗∗∗P < 0.001. PGC1ɑ, peroxisome proliferator-activated receptor γ coactivator α; TFAM, mitochondrial transcription factor A; TOMM20, translocase of the outer mitochondrial membrane complex subunit 20; PINK1, tensin homolog induced kinase 1; LC3B, microtubule-associated protein 1 light chain 3B; p62, ubiquitin-binding protein sequestosome 1; IL8, interleukin 8; p-YAP1, phosphorylated YAP1.

In the GSE104954 dataset (comprising N = 21 control, and N = 17 DKD samples), a notable reduction in YAP1 transcripts within human diabetic kidneys was observed, as depicted in Fig. 1A and B. Furthermore, our analysis revealed downregulation of genes associated with mitobiogenesis (*mitochondrial transcription factor A [TFAM]*, and *translocase of the outer mitochondrial membrane complex subunit 20 [TOMM20]*) and mitophagy (*phosphatase and tensin homolog induced kinase 1 [PINK1]*) in diabetic kidneys, while genes linked to disrupted autophagy (*ubiquitin-binding protein sequestosome 1 [p62]*) exhibited significantly elevated expression levels in the diabetic kidney samples compared to controls (Fig. 1A and B). Additionally, the receiver operating characteristic curve (ROC) analysis demonstrated the potential utility of these significantly altered genes as effective individual markers for distinguishing DKD, as illustrated in Fig. 1C. More importantly, when these markers were combined into a comprehensive predictive model for the occurrence of DKD, the resulting area under the ROC curve reached an impressive 0.93, indicating excellent predictive performance of this combined model (Fig. 1D).

In line with the results from the microarray dataset, cultured human renal proximal tubule cells (HK-2) treated with palmitic acid (PA) and high glucose (HG) resulted in the inactivation of YAP1 (as evidenced by decreased YAP1, while increased p-YAP1), mitobiogenesis-related molecules (peroxisome proliferator-activated receptor γ coactivator α [PGC1α], TFAM and TOMM20), and mitophagy-related molecules (PINK1 and Parkin), concomitant with dysregulated autophagy flux (as evidenced by increased microtubule-associated protein 1 light chain 3B-II [LC3B-II] and p62) [30] and elevated expression of the proinflammatory marker interleukin 6 (IL6), as outlined in Fig. 1E and F. The transcript levels displayed a similar trend to the observed protein levels, as illustrated in Fig. 1H. Besides, transmission electron microscopy (TEM) analysis found damaged mitochondria with swollen matrix and collapsed cristae within HK-2 cells exposed to PA and HG (Fig. 1G). Cultured HK-2 cells exposed to PA and HG displayed lower mitochondrial membrane potential, but higher level of mitochondrial reactive oxygen species (mtROS) (Fig. 1I–L).

**Fig. 2:**
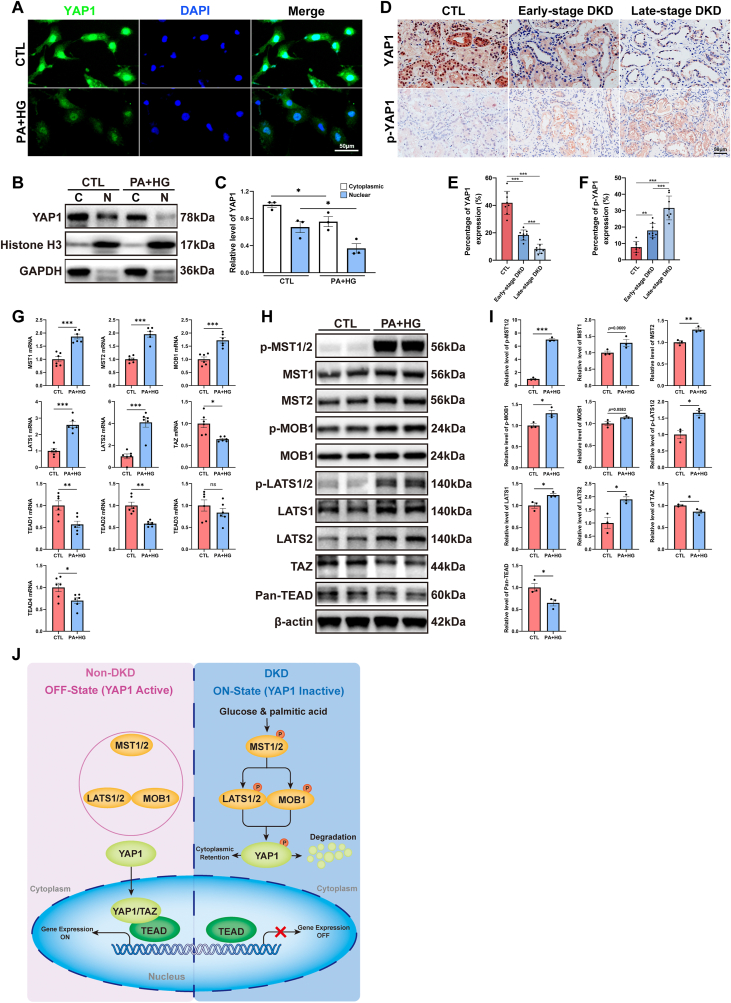
Hippo signaling pathway was activated in tubule cells under the context of diabetic kidney disease (DKD). (**A**) Representative images of the immunofluorescence staining for yes-associated protein 1 (YAP1) in cultured HK-2 cells with or without palmitic acid and high glucose (PA + HG) treatment. Bar = 50 μm. (**B**, **C**) Representative Western blots and quantification of YAP1 in cytoplasmic and nuclear fraction of HK-2 cells exposed to PA + HG. (**D**) Representative images of immunohistochemistry staining for YAP1 and p-YAP1 in kidney sections from control donors, early-stage DKD patients, and late-stage DKD patients. Bar = 50 μm. (**E**, **F**) Quantitative assessment for percentage of YAP1 and p-YAP1 expression (n = 8 for each group). (**G**) Transcripts of genes involved in Hippo signaling pathway were measured by quantitative real-time polymerase chain reaction in cultured HK-2 cells with or without PA + HG treatment. (**H**, **I**) Representative Western blots and quantification of key molecules involved in Hippo signaling pathway in HK-2 cells exposed to PA + HG. (**J**) Schematic diagram showing the activation of Hippo signaling pathway and the ultimately degraded YAP1 in tubule cells under the context of DKD. Data are expressed as the mean ± SEM. All experiments were repeated at least three times. ∗P < 0.05; ∗∗P < 0.01; ∗∗∗P < 0.001. MST1, serine/threonine kinase 4; MST2, serine/threonine kinase 3; p-MST1/2, phosphorylated MST1/2; MOB1, MOB kinase activator 1; p-MOB1, phosphorylated MOB1; LATS1, large tumor suppressor kinase 1; LATS2, large tumor suppressor kinase 2; p-LATS1/2, phosphorylated LATS1/2; TAZ, Tafazzin; TEAD, TEA domain transcription factor; DAPI, 4′,6-diamidino-2-phenylindole; p-YAP1, phosphorylated YAP1.

Using immunofluorescence and immunoblot, we further illustrated the decreased cytoplasmic and nuclear expression of YAP1, as well as impeded translocation of YAP1 from the cytoplasm to the nucleus in cultured HK-2 cells subjected to PA and HG treatment (Fig. 2A–C). Moreover, immunohistochemistry of tubulointerstitial compartment of kidney sections confirmed a reduction of YAP1 protein and an increase in S127 phosphorylated YAP1 from patients of biopsy-approved DKD, particularly in the late-stage DKD subjects (Fig. 2D–F), suggesting augmented YAP1 protein degradation in diabetic tubule cells. Additionally, quantitative real-time polymerase chain reaction (qRT-PCR) and immunoblots results revealed the significantly activated Hippo signaling pathway in HK-2 cells treated with PA and HG (Fig. 2G–I). Collectively, our study, integrating results from bioinformatic analysis of transcriptomics data, *in vitro* investigations, and pathological analyses of biopsy-approved DKD kidney sections, unveils the inactivation of YAP1 at both transcription and post-translation levels under the context of DKD. Particularly, at the post-translation level, our findings unmasked the activation of the Hippo signaling pathway in tubule cells affected by DKD, thus leading to a significantly decreased accumulation of the key downstream mediator YAP1 in the nucleus where it typically binds to TEA domain transcription factors and drives the expression of target genes (Fig. 2J).

### 3.2 Renal tubule cell-specific *Yap1* deletion did not affect the development of diabetes

**Fig. 3:**
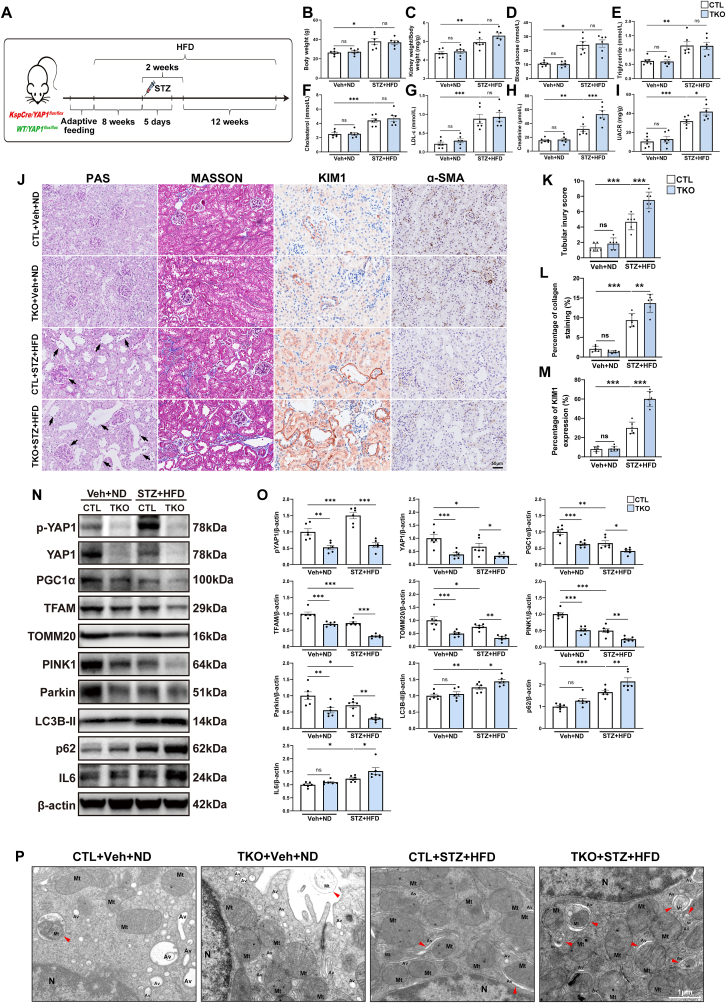
Renal tubule cell-specific *yes-associated protein 1 (Yap1)* deletion aggregated kidney injury via disrupting mitochondrial quality control (MQC) in diabetic kidney disease (DKD) mice. (**A**) The schematic diagram of *in vivo* experiment. (**B–I**) Body weight, kidney weight/body weight, blood glucose, serum triglyceride, cholesterol, low-density lipoprotein cholesterol (LDL-c), and creatinine and urinary albumin-to-creatinine ratio (UACR) in the indicated mouse groups. (**J**) Representative images of histologic images of Periodic acid-Schiff (PAS) staining, Masson's trichrome (MASSON) staining, as well as immunohistochemistry staining for kidney injury molecule-1 (KIM1) and ɑ-smooth muscle actin (ɑ-SMA) in kidney sections. Black arrows indicated expanded mesangial expansion and tubular atrophy, dilation, or brush border loss. Bar = 50 μm. (**K-M**) Quantitative assessment for tubular injury score, percentage of collagen staining and percentage of KIM1 expression. (**N, O**) Representative Western blots and quantification of p-YAP1, YAP1, MQC-related mediators, and interleukin 6 (IL6) in renal cortex from indicated mouse groups. (**P**) Representative transmission electron microscopy images of mitochondria with abnormal morphology in tubule cells from indicated mouse groups. Damaged mitochondria were observed with matrix swelling and collapsed cristae. An early phase of mitophagy was seen as the formation of double membrane, wrapped around a mitochondrion. The red arrowheads indicate autophagic vacuoles containing damaged mitochondria, or autophagosomes surrounded by a double-membrane with undigested damaged mitochondria inside. Bar = 1 μm. Mt, mitochondrion; Av, autophagic vacuole; N, nucleus. Data are expressed as the mean ± SEM. ∗P < 0.05; ∗∗P < 0.01; ∗∗∗P < 0.001 (n = 6 for each group). ND, normal diet; HFD, high-fat diet; STZ, streptozotocin; TKO, tubule cell-specific knockout; Veh, vehicle; PGC1ɑ, peroxisome proliferator-activated receptor γ coactivator α; TFAM, mitochondrial transcription factor A; TOMM20, translocase of the outer mitochondrial membrane complex subunit 20; PINK1, tensin homolog induced kinase 1; p62, ubiquitin-binding protein sequestosome 1; LC3B, microtubule-associated protein 1 light chain 3B; p-YAP1, phosphorylated YAP1.

To dig deeper into the role of YAP1 in the pathogenesis of DKD, we generated mice with renal tubule cell-specific *Yap1* knockout mice (TKO, *KspCre*/*Yap1*^*flox/flox*^), with their littermates lacking the *KspCre* allele serving as control mice (CTL, *WT/Yap1*^*flox/flox*^). The genotyping results are provided in the Supplementary Fig. S1, and the experimental design is detailed in Fig. 3A. Treatment with STZ + HFD led to a significant increase in body weight, kidney index (as calculated by kidney weight/body weight), blood glucose, serum triglyceride, cholesterol, low-density lipoprotein cholesterol (LDL-c), creatinine, and urinary albumin-to-creatinine ratio (UACR) when compared to mice treated with ND. Mice subjected to STZ + HFD exhibited a nearly 1.4-fold increase in body weight, a nearly 2-fold increase in blood glucose, serum creatinine, cholesterol, and triglyceride, a nearly 3-fold increase in UACR, and a nearly 4-fold increase in LDL-c (Fig. 3B–I). It's noteworthy that TKO mice and their CTL littermates were normal in size and did not display any gross physical or behavioral abnormalities, and there were no discernible differences in kidney size or morphology (Supplementary Fig. S2). In TKO mice, YAP1 protein levels in the kidney were significantly reduced when compared to those in CTL mice (Fig. 3N and O). No significant differences in body weight and kidney index were observed between TKO and CTL mice under either ND or HFD conditions (Fig. 3B and C). Blood glucose and serum lipid profiles including serum triglyceride, cholesterol, and LDL-c (Fig. 3D–G) showed similar elevations in both TKO mice and CTL mice subjected to HFD, indicating that tubular YAP1 deficiency does not significantly influence the metabolic profiles in diabetic mice.

### 3.3 Renal tubule cell-specific *Yap1* deletion aggregated kidney injury in DKD mice

The diabetes-induced elevation in serum creatinine and UACR levels observed in CTL mice was notably exacerbated following the deletion of tubular *Yap1* (Fig. 3H and I). Periodic acid Schiff (PAS) staining vividly depicted the heightened kidney injury in TKO mice induced by diabetes, evident through elevated tubular injury score (Fig. 3J and K**)**. Masson's trichrome staining further underscored the exacerbated interstitial fibrosis, characterized by increased collagen production (Fig. 3J and L**)**. The upregulated protein expression of tubular injury marker, namely kidney injury molecule-1 (KIM1), was further intensified by the deletion of tubular *Yap1* (Fig. 3J and M**)**. The fibrotic marker ɑ-smooth muscle actin (ɑ-SMA) exerted no difference between neither diabetic and lean control mice nor TKO and CTL mice (Fig. 3J). These collective findings strongly imply that *Yap1* deficiency eradicated the kidney's innate resilience to diabetes-induced injury.

### 3.4 Renal tubule cell-specific *Yap1* deletion disrupted MQC in DKD mice

To elucidate the mechanisms underlying the exacerbated tubular impairment observed in TKO diabetic mice, we isolated the renal cortex from diabetic and non-diabetic mice to scrutinize the expression of p-YAP1, YAP1 and MQC-related molecules. Consistent with the findings from *in vitro* study, immunoblots of renal cortex confirmed a reduction of YAP1 protein and an increase in S127 phosphorylated YAP1 from diabetic mice (Fig. 3N and O), indicating increased YAP1 protein degradation in diabetic renal cortex. Besides, our analysis unveiled a significant downregulation of mitobiogenesis-related mediators (PGC1α, TFAM, and TOMM20) and mitophagy-related mediators (PINK1 and Parkin), but a significant upregulation of dysregulated autophagy-related mediator (LC3B-II and p62) [30] and proinflammatory mediator (IL6) at protein levels in TKO mice when compared to CTL mice (Fig. 3N and O). Furthermore, we examined the ultrastructural changes in mitochondria within tubule cells from diabetic and non-diabetic mice using TEM. In non-diabetic mice, the tubule cells displayed a population of healthy, normal-appearing mitochondria. Importantly, tubule cells from non-diabetic mice demonstrated numerous autophagic vacuoles, accompanied by autophagosome and autolysosome, indicating the existence of a smooth and integrative process of mitophagy/autophagy flux (Fig. 3P). In contrast, mice subjected to HFD in combination with intraperitoneal STZ injection, particularly the TKO diabetic mice, exhibited an accumulation of damaged mitochondria with swollen matrix and collapsed cristae, a decrement of autophagic vacuoles, as well as fusion of autophagic vacuoles with abnormal mitochondria within tubule cells, indicating the accumulated autophagosomes, dysregulated MQC with morphological impairment and blocked mitophagy/autophagy flux in DKD pathogenesis (Fig. 3P).

### 3.5 C-X-C motif chemokine ligand 1 (CXCL1) expression and macrophage infiltration were enhanced in human diabetic kidney

**Fig. 4:**
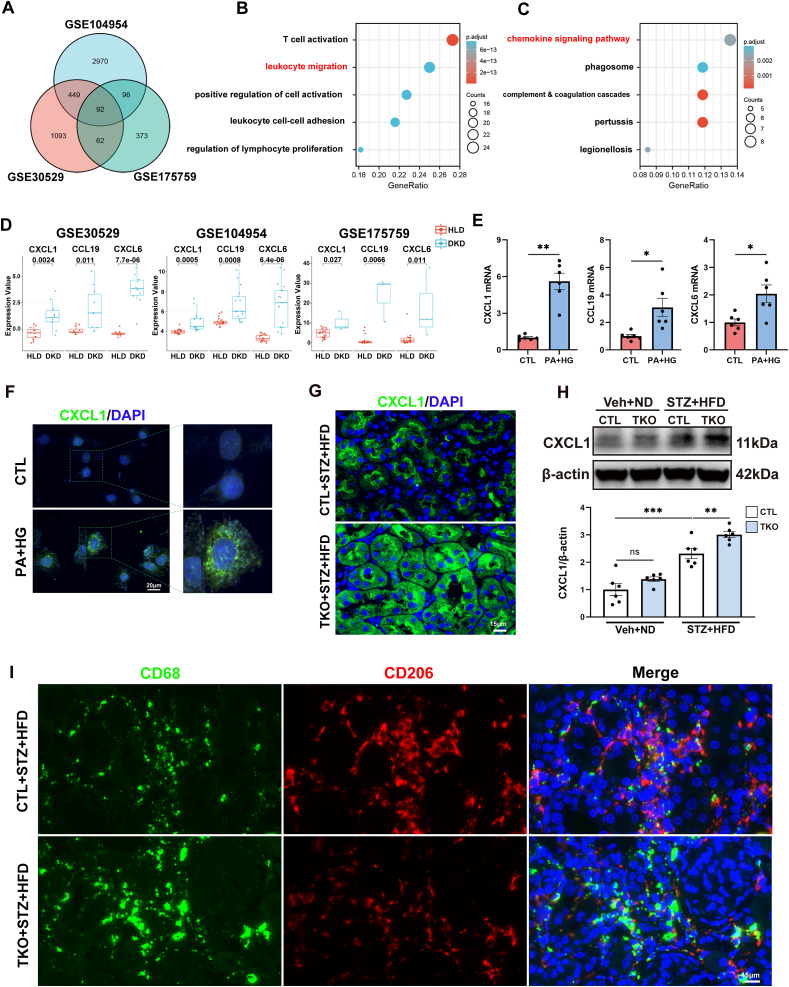
Renal tubule cells-specific *yes-associated protein 1 (Yap1)* deletion boosted CXCL1 secretion and affected macrophage polarization in diabetic kidney. (**A**) Overlapped differentially expressed genes (DEGs) recognized among GSE104954, GSE175759 and GSE30529. (**B**) Gene ontology enrichment analyses of overlapped DEGs. (**C**) Kyoto Encyclopedia of Genes and Genomes (KEGG) enrichment analysis of overlapped DEGs. (**D**) Renal expression of chemokines C-X-C motif chemokine ligand 1 (CXCL1), C–C motif chemokine ligand (CCL19) and CXCL6 in 3 selected GSE datasets (GSE30529, GSE104954, and GSE175759). (**E**) Transcripts of *CXCL1*, *CCL19* and *CXCL6* were measured by quantitative real-time polymerase chain reaction in cultured HK-2 cells with or without palmitic acid and high glucose treatment (PA + HG). (**F**) Representative images of the immunofluorescence staining for CXCL1 in cultured HK-2 cells with or without PA + HG treatment. Bar = 20 μm. (**G**) Representative images of the immunofluorescence staining for CXCL1 in kidney sections from indicated mouse groups. Bar = 15 μm. (**H**) Representative Western blots and quantification of CXCL1 in renal cortex from indicated mouse groups. (**I**) Representative images of the immunofluorescence staining for CD68 and CD206 in kidney sections from indicated mouse groups. Bar = 15 μm. Data are expressed as the mean ± SEM. ∗P < 0.05; ∗∗P < 0.01; ∗∗∗P < 0.001 (n = 6 for each group). ND, normal diet; HFD, high-fat diet; STZ, streptozotocin; TKO, tubule cell-specific knockout; Veh, vehicle; DAPI, 4′,6-diamidino-2-phenylindole.

To investigate the potential link between *Yap1* deficiency-induced disruption of MQC in tubule cells and the alternated immune status within diabetic mouse kidneys, we first conducted an ongoing bioinformatic analysis employing microarray datasets relevant to human DKD. In addition to GSE104954, we retrieved two additional microarray datasets (GSE30529 and GSE175759) originating from the tubulointerstitial compartments of human diabetic kidneys via the GEO database. An analysis of overlapping differentially expressed genes (DEGs) among these three datasets unveiled a total of 92 common DEGs shared between GSE104954, GSE30529, and GSE175759 (Fig. 4A and Supplementary Table S5). Subsequent gene ontology (GO) analysis illuminated the top five enriched GO terms, including T cell activation, leukocyte migration, positive regulation of cell activation, leukocyte cell-cell adhesion, and regulation of lymphocyte proliferation (Fig. 4B and Supplementary Table S6). Concurrently, the Kyoto Encyclopedia of Genes and Genomes (KEGG) pathway analysis uncovered the top five signaling pathways, including the chemokine signaling pathway, phagosome, complement and coagulation cascades, pertussis, and legionellosis, underscoring the pivotal role of chemokine-mediated biological processes in the progression of DKD (Fig. 4C). Correspondingly, among the commonly shared DEGs across the three datasets, three chemokines (*C-X-C motif chemokine ligand 1 [CXCL1], CXCL6,* and *C–C motif chemokine ligand [CCL19]*) derived from the KEGG chemokine signaling pathway exhibited significantly elevated expression in diabetic kidneys (Fig. 4D and Supplementary Table S7), in line with results from cultured HK-2 cells exposed to PA and HG (Fig. 4E and F). These findings implicated the active involvement of tubule cells-derived chemokines, particularly CXCL1, and chemokine signaling pathways in fostering immune cell activation within diabetic kidneys.

### 3.6 Renal tubule cell-specific *Yap1* deletion boosted CXCL1 secretion and affected macrophages polarization in DKD mice

**Fig. 5:**
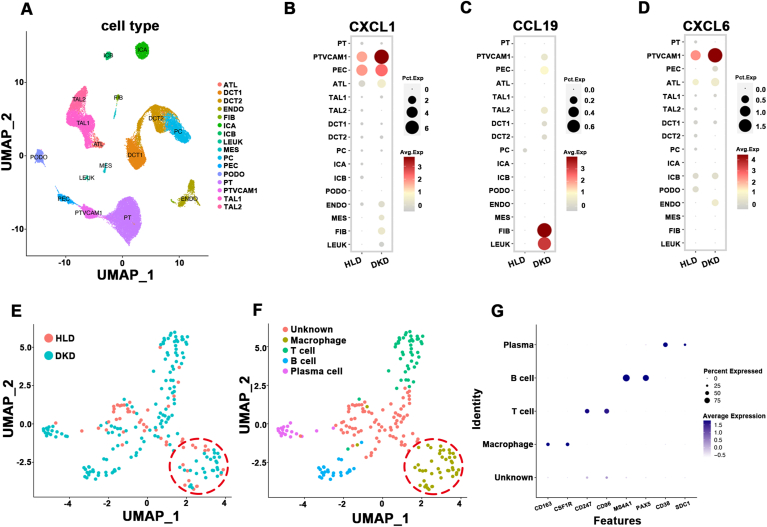
C-X-C motif chemokine ligand 1 (CXCL1) expression and macrophage infiltration were enhanced in human diabetic kidney. (**A**) The identified cell clusters by t-distributed Stochastic Neighbor Embedding (t-SNE) analysis in single nucleus RNA-sequencing (snRNA-seq) dataset merged by GSE131882, GSE151302 and GSE195460. PT, proximal convoluted tubule; PTVCAM1, vascular cell adhesion molecule 1 (VCAM1)-positive proximal convoluted tubule; PEC, parietal epithelial cells; ATL, ascending thin limb; TAL, thick ascending limb; DCT, distal convoluted tubule; PC, principal cells; ICA, type A intercalated cells; ICB, type B intercalated cells; PODO, podocyte; ENDO, endothelial cells; MES, mesangial cells; FIB, fibroblasts; LEUK, leukocytes. (**B-D**) Bubble charts show the expression of chemokine genes (*CXCL1*, *CCL19*, and *CXCL6*) in tubule cells from snRNA-seq dataset merged by GSE131882, GSE151302 and GSE195460. (**E**) The distribution of immune cells in kidney from snRNA-seq dataset merged by GSE131882, GSE151302 and GSE195460. Red dashed circle shows macrophage. (**F**) The distribution of macrophage, T cell, B cell and plasma cell in kidney from snRNA-seq dataset merged by GSE131882, GSE151302 and GSE195460. Red dashed circle shows macrophage. (**G**) The expression patterns of macrophage, T cell, B cell and plasma cell. UMAP, uniform manifold approximation and projection; CXCL1, C-X-C motif chemokine ligand 1; CCL19, C–C motif chemokine ligand; CXCL6, C-X-C motif chemokine ligand 6.

We subsequently substantiate whether the dysregulation of MQC resulting from *Yap1* deficiency influenced the immune status within the diabetic kidney. Immunofluorescence analyses revealed a substantial increase in the expression of chemokine CXCL1 within the tubule cells of diabetic kidneys (Supplementary Fig. S3). Remarkably, the expression level of CXCL1 was significantly elevated in the tubule cells of TKO diabetic mice when compared to CTL diabetic mice (Fig. 4G and H). Furthermore, immunofluorescence examination unveiled a noticeable increase in the infiltration of macrophages in diabetic mice, which was evident through the significantly augmented expression of the M1 macrophage marker CD68 and the M2 macrophage marker CD206 within the diabetic kidney (Supplementary Fig. S4). Intriguingly, *Yap1* deficiency appeared to aggregate the infiltration of M1 macrophages by suppressing M2 macrophage polarization in TKO diabetic mice in comparison to CTL diabetic mice (Fig. 4I). To further validate the insights gained from microarray datasets and diabetic mice kidneys, we integrated human diabetic kidney-related single nucleus RNA-sequencing (snRNA-seq) datasets, namely GSE195460, GSE151302, and GSE131882, in an effort to probe the dynamics of chemokines (specifically CXCL1, CXCL6 and CCL19) and immune cell infiltration. Notably, among the tubule cells, we identified a subset characterized by vascular cell adhesion molecule 1 (VCAM1) positivity (Fig. 5A). Consistently, the snRNA-seq analysis revealed that CXCL1 and CXCL6 were primarily expressed by VCAM1-positive tubule cells, with a marked increase in their expression in these cells within the diabetic kidney (Fig. 5B and D). In contrast, CCL19 was predominantly expressed by fibroblasts and leukocytes (Fig. 5C). Furthermore, our analysis demonstrated a significant increase in the number of infiltrating immune cells within the diabetic kidney (Fig. 5E). In accordance with the findings from microarray analysis and diabetic mice kidneys, this increase was particularly pronounced among macrophages, T cells, B cells, and plasma cells (Fig. 5F and G). These findings via bioinformatic analyses align with the results obtained from *Yap1*-deficient diabetic mice, underscoring that *Yap1* deficiency-induced perturbations in MQC within tubule cells may potentially stimulate the secretion of CXCL1 from these cells, thereby contributing to the polarization of macrophages within the diabetic microenvironment.

### 3.7 Yap1 activation maintained MQC homeostasis and counteracted diabetes-induced kidney injury

**Fig. 6:**
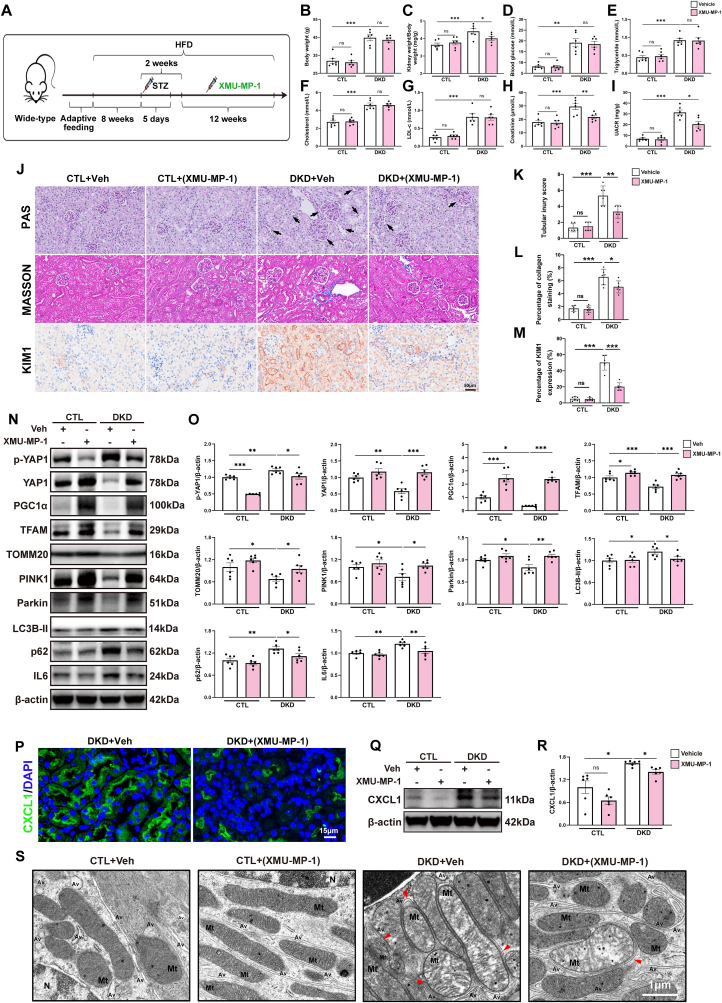
*Yes-associated protein 1 (Yap1)* activation counteracted diabetes-induced kidney injury via maintaining mitochondrial quality control (MQC) homeostasis in diabetic kidney. (**A**) The schematic diagram of *in vivo* experiment. (**B–I**) Body weight, kidney weight/body weight, blood glucose, serum triglyceride, cholesterol, low-density lipoprotein cholesterol (LDL-c), and creatinine and urinary albumin-to-creatinine ratio (UACR) in the indicated mouse groups. (**J**) Representative images of histologic images of Periodic acid-Schiff (PAS) staining, Masson's trichrome (MASSON) staining, as well as immunohistochemistry staining for kidney injury molecule-1 (KIM1) in kidney sections. Black arrows indicated expanded mesangial expansion and tubular atrophy, dilation, or brush border loss. Bar = 50 μm. (**K-M**) Quantitative assessment for tubular injury score, percentage of collagen staining and percentage of KIM1 expression. (**N, O**) Representative Western blots and quantification of p-YAP1, YAP1, MQC-related mediators, and interleukin 6 (IL6) in renal cortex from indicated mouse groups. (**P**) Representative images of the immunofluorescence staining for C-X-C motif chemokine ligand 1 (CXCL1) in kidney sections from indicated mouse groups. Bar = 15 μm. (**Q-R**) Representative Western blots and quantification of CXCL1 in renal cortex from indicated mouse groups. (**S**) Representative transmission electron microscopy images of mitochondria with abnormal morphology in tubule cells from indicated mouse groups. Damaged mitochondria were observed with matrix swelling and collapsed cristae. An early phase of mitophagy was seen as the formation of double membrane, wrapped around a mitochondrion. The red arrowheads indicate autophagic vacuoles containing damaged mitochondria, or autophagosomes surrounded by a double-membrane with undigested damaged mitochondria inside. Bar = 1 μm. Mt, mitochondrion; Av, autophagic vacuole; N, nucleus. Data are expressed as the mean ± SEM. ∗P < 0.05; ∗∗P < 0.01; ∗∗∗P < 0.001 (n = 6 for each group). HFD, high-fat diet; STZ, streptozotocin; DKD, diabetic kidney disease; Veh, vehicle; PGC1ɑ, peroxisome proliferator-activated receptor γ coactivator α; TFAM, mitochondrial transcription factor A; TOMM20, translocase of the outer mitochondrial membrane complex subunit 20; PINK1, tensin homolog induced kinase 1; LC3B, microtubule-associated protein 1 light chain 3B; p62, ubiquitin-binding protein sequestosome 1; DAPI, 4′,6-diamidino-2-phenylindole; p-YAP1, phosphorylated YAP1.

We investigated the therapeutic potential of the *Yap1* activator, XMU-MP-1, in DKD mice model to assess its impact on diabetic mice. The experimental design is illustrated in Fig. 6A. Diabetic mice were subjected to a 12-week treatment regimen with either XMU-MP-1 or an equivalent volume of vehicle (dimethyl sulfoxide, DMSO). Notably, there were no discernible differences in body weight between the (XMU-MP-1)-treated mice and those receiving DMSO (Fig. 6B). Similarly, an evaluation of blood glucose and serum lipid profiles (including triglyceride, cholesterol, and LDL-c) within the diabetic groups revealed no significant distinctions between the (XMU-MP-1)-treated and DMSO-treated mice (Fig. 6D–G). Nevertheless, a notable reduction of kidney index, serum creatinine, and UACR in (XMU-MP-1)-treated mice in comparison to DMSO-treated mice was observed (Fig. 6C–H and I). Additionally, we observed a reduction in pathological kidney injury in the (XMU-MP-1)-treated mice compared to the DMSO-treated mice (Fig. 6J), evident through reduced tubular injury score by PAS staining (Fig. 6K), weakened collagen production by Masson's trichrome staining (Fig. 6L), and decreased expression of KIM1 by immunohistochemistry (Fig. 6M). Furthermore, gene set enrichment analysis from RNA-sequencing data of diabetic mice kidney revealed that Hippo signaling pathway (Supplementary Fig. S5), chemokine signaling pathway, and cell activation involved in immune response were positively associated with diabetes, while mitochondrion organization was negatively associated with diabetes (Supplementary Fig. S6). By contrast, administration of XMU-MP-1 partly suppressed the irritation of chemokine signaling pathway, and organ or tissue specific immune response, but preserved mitochondrion organization within diabetic mice kidney (Supplementary Fig. S6). Additionally, in the (XMU-MP-1)-treated diabetic mice, YAP1, key mediators of mitobiogenesis (PGC1α, TFAM and TOMM20) and mitophagy (PINK1 and Parkin) exhibited preserved protein levels (Fig. 6N and O). The protective influence of XMU-MP-1 towards renal MQC resulted in smooth mitochondria/autophagy flux and ameliorated kidney inflammation, as evidenced by decreased protein expression of LC3B-II, p62 and IL6 (Fig. 6N and O). Furthermore, immunofluorescence and Western blot illustrated the beneficial impact of XMU-MP-1 in preventing synthesis and secretion of CXCL1 within tubule cells (Fig. 6P–R). Consistently, CIBERSORT analysis of RNA-sequencing data of diabetic mice kidney displayed the decreased proportion of M1 macrophage infiltration within kidney microenvironment by administration of XMU-MP-1 (Supplementary Fig. S7). Moreover, TEM analysis exhibited a reduction of damaged mitochondria with swollen matrix and collapsed cristae, an increment of autophagic vacuoles, as well as diminished fusion of autophagic vacuoles with anormal mitochondria within tubule cells from (XMU-MP-1)-treated diabetic mice, suggesting a decrease in the accumulation of autophagosomes, improved MQC with morphological integrity and smooth mitophagy/autophagy flux by XMU-MP-1 treatment (Fig. 6S). Taken together, these data strongly suggest that XMU-MP-1 may safeguard the kidney from diabetes-induced injury by preserving tubular MQC homeostasis, and subsequently suppress macrophage activation and renal inflammation by preventing CXCL1 production.

### 3.8 YAP1 maintained MQC homeostasis in HK-2 cells

**Fig. 7:**
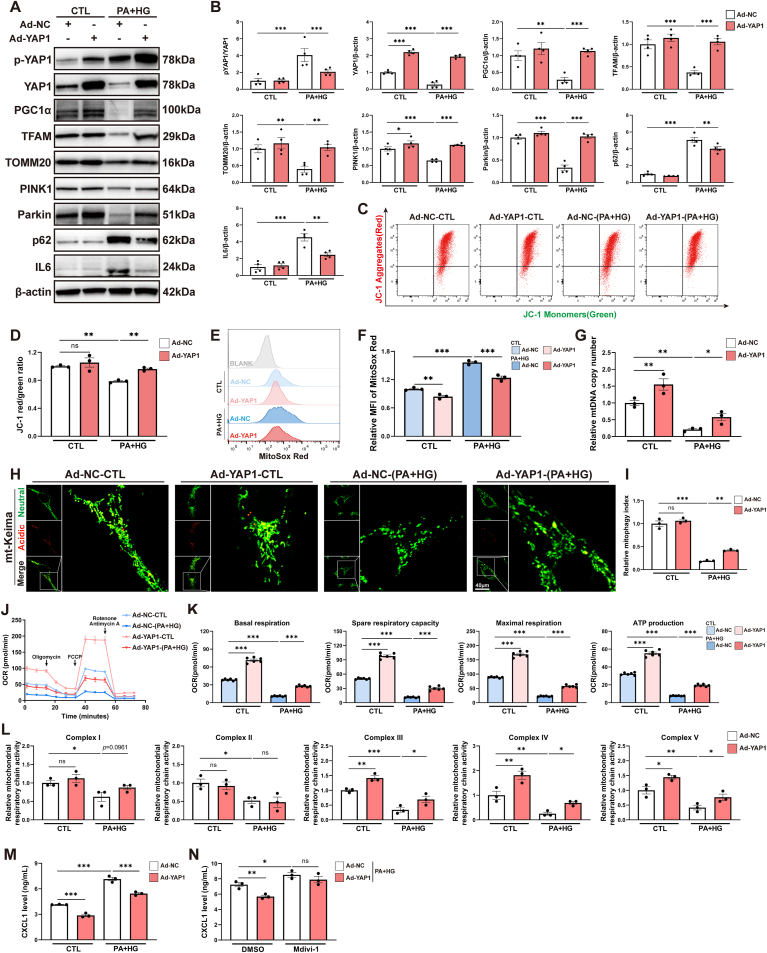
Yes-associated protein 1 (YAP1) maintained mitochondrial quality control (MQC) homeostasis in cultured HK-2 cells. (**A, B**) Representative Western blots and quantification of p-YAP1, YAP1, MQC-related mediators, and interleukin 6 (IL6) in adenovirus (Ad)-infected HK-2 cells with or without palmitic acid and high glucose (PA + HG) treatment. (**C, D**) Representative plots and quantification of flow cytometry analysis for JC-1 staining in Ad-infected HK-2 cells with or without PA + HG treatment. The JC-1 red/green fluorescence ratio was calculated to represent mitochondrial membrane potential. (**E, F**) Representative plots and quantification of flow cytometry analysis for MitoSox Red staining in Ad-infected HK-2 cells with or without PA + HG treatment. The mean fluorescence intensity of MitoSox Red was calculated to represent mitochondrial reactive oxygen species level. (**G**) The relative mitochondrial DNA (mtDNA) copy numbers in Ad-infected HK-2 cells with or without PA + HG treatment were measured by qPCR. (**H**) Representative confocal images of mt-Keima in Ad-infected HK-2 cells with or without PA + HG treatment. Confocal microscopy was analyzed to detect the mt-Keima located in mitochondria (mitochondria at neutral pH, green fluorescence) and the mt-Keima delivered to lysosomes (mitochondria at acidic pH, red fluorescence). The zoom images were magnified from boxed areas in overlay images. Bar = 40 μm. (**I**) Quantification of mitophagy index by mt-Keima imaging. Mitophagy index was determined by analyzing the ratio of red/green fluorescence. (**J**) Real-time measurements of the oxygen consumption rate (OCR) in Ad-infected HK-2 cells with or without PA + HG treatment via Seahorse XF96. After basal OCR was obtained, oligomycin (1.5 μM) was added to obtain ATP-linked OCR. Then, the uncoupler FCCP (1 μM) was added to obtain maximal OCR. Finally, none-mitochondrial OCR was obtained after adding Antimycin A + rotenone (0.5 μM each) to inhibit the electron transport chain. Mitochondrial spare respiratory capacity was calculated by subtracting basal respiration from maximal respiratory capacity. Each point in the lines represents the average measurements of six different wells. (**K**) OCR results from the Seahorse analysis of the mitochondrial respiration parameters: basal respiration, spare respiratory capacity, maximal respiration, and ATP production. (**L**) Mitochondrial respiratory chain complex enzyme activities in Ad-infected HK-2 cells with or without PA + HG treatment. (**M**) ELISA of CXCL1 level for Ad-infected HK-2 cells with or without PA + HG treatment. (**N**) ELISA of CXCL1 level for Ad-infected HK-2 cells with or without mitophagy inhibitor Mdivi-1 treatment. Data are expressed as the mean ± SEM. All experiments were repeated at least three times. ∗P < 0.05; ∗∗P < 0.01; ∗∗∗P < 0.001. NC, negative control; PGC1ɑ, peroxisome proliferator-activated receptor γ coactivator α; TFAM, mitochondrial transcription factor A; TOMM20, translocase of the outer mitochondrial membrane complex subunit 20; PINK1, tensin homolog induced kinase 1; p62, ubiquitin-binding protein sequestosome 1; IL6, interleukin 6; CXCL1, C-X-C motif chemokine ligand 1; p-YAP1, phosphorylated YAP1; FCCP, carbonyl cyanide-p-trifluoromethoxyphenylhydrazone; ELISA, enzyme-linked immunosorbent assay.

We conducted a comprehensive investigation into the pivotal role and mechanism of YAP1 in regulating MQC homeostasis using an adenovirus knock-in approach in cultured HK-2 cells. The successful adenovirus knock-in resulted in a substantial upregulation of YAP1 and p-YAP1 expression in HK-2 cells, as depicted in Fig. 7A and B. Remarkably, we observed a reversal of the suppressed protein levels of MQC-related mediators, including PGC1α, TFAM, TOMM20, PINK1, and Parkin, when YAP1 was highly expressed in HK-2 cells exposed to PA and HG, as illustrated in Fig. 7A and B. Furthermore, the PA and HG treatment significantly disrupted mitochondrial membrane potential, increased mtROS production, reduced mtDNA copy numbers, and inhibited mitophagy in cultured HK-2 cells (Fig. 7C–I). In contrast, all these detrimental effects were mitigated when YAP1 was highly expressed in HK-2 cells exposed to PA and HG, as illustrated in Fig. 7C–I. Additionally, a bioenergetic profile was established by administering electron transport chain inhibitors to assess mitochondrial oxygen consumption rate (OCR) in various respiratory states (Fig. 7J). Fig. 7K illustrated that treatment with PA and HG significantly reduced basal respiration, spare respiratory capacity, maximal respiration, and ATP production. Conversely, there was an increase in these parameters when YAP1 was overexpressed in HK-2 cells compared to the adenovirus knock-in negative control. As depicted in Fig. 7L and Supplementary Fig. S8, PA + HG treatment resulted in a decrease in both the expression of subunits and the enzymatic activity of mitochondrial complexes I, II, III, IV, and V. Notably, YAP1 overexpression partially reversed the suppression of subunit expression and enzymatic activity for complexes III, IV, and V. However, while YAP1 restored subunit expression in complexes I and II, it did not recover their enzymatic activity. This discrepancy between the enzymatic activity assays and Western blot detection of subunits for complexes I and II may stem from several factors, including post-translational modifications [[31], [32], [33], [34], [35]], the specific contributions of individual subunits to enzymatic function [36,37], and potential issues with subunit stoichiometry and assembly defects [38,39]. Furthermore, a noticeable reversal of the elevated CXCL1 concentration in the culture medium was observed when YAP1 was highly expressed in HK-2 cells exposed to PA and HG (Fig. 7M). Treatment with the mitophagy inhibitor Mdivi-1 exacerbated CXCL1 production and abolished the reversal of CXCL1 elicited by YAP1 overexpression in HK-2 cells exposed to PA and HG (Fig. 7N). Importantly, the upregulation of YAP1 in HK-2 cells did not compensate for the increased susceptibility to elevated CXCL1 production induced by mitophagy inhibition through Mdivi-1 pretreatment, suggesting a potential causal relationship between dysregulated MQC and CXCL1 generation in tubule cells. Collectively, our findings strongly support the notion that YAP1 possesses the capacity to modulate the expression of MQC-related molecules, and preserves PGC1α-mediated mitobiogenesis and PINK1/Parkin-mediated mitophagy, thus contributing to maintaining the MQC homeostasis and subsequently suppressing CXCL1 production in HK-2 cells.

## 4 Discussion

One of the central findings of our study is the observed inactivation of YAP1 in tubule cells among individuals with diabetes and its accompanying impact on maintaining MQC homeostasis. The role of YAP1 in kidney disease remains a topic of ongoing debate, with existing discrepancies likely stemming from variations in experimental animal models and the specific renal compartments under investigation [26,28,[40], [41], [42], [43]]. For instance, although existing evidence suggests that YAP1 plays a crucial role in podocyte differentiation, cell survival, maintenance of the specialized structure [[44], [45], [46]], and podocyte-specific deletion of YAP1 accelerated renal injury in diabetes, conflicting findings have emerged. Some studies have reported heightened YAP1 activity in podocytes during diabetes [40,47], noting a close correlation between increased expression of podocyte YAP1 and more severe renal pathological damage during DKD progression [40]. Similarly, elevated YAP1 levels in tubule cells have been previously reported, and inhibiting the YAP1 activity through pharmacological or genetic means has shown promising results in ameliorating renal fibrosis, inflammation, and oxidative stress in experimental type 1 diabetes-related DKD models [26,48]. Conversely, YAP1 was found to be suppressed in tubule cells from adriamycin-induced kidney injury mice model [49], and preserved YAP1 plays a crucial role in mediating tubule cell regeneration during kidney recovery [50,51]. The contrasting effects observed for YAP1 underscore its complex role and mechanism across various renal compartments, different stages of kidney disease progression, and diverse contexts of kidney disease types. Our current study for the first time demonstrated the consistent inactivation of YAP1 signaling in biopsy-approved human diabetic kidney, cultured HK-2 cells exposed to HG and PA, and tubule cells from DKD mice induced by a HFD plus STZ injection.

Our investigation further unveiled the robust diagnostic accuracy of a comprehensive predictive model consisted of YAP1 and MQC-related genes in identifying DKD, which underscores the value of these markers in early DKD detection. To elucidate the mechanistic link between YAP1 inactivation and DKD progression, we turned to experimental models. Tubule ell-specific knockout of *Yap1* exacerbated kidney injury in diabetic mice, emphasizing the protective role of YAP1 within tubule cells and its contribution to mitigating kidney injury in the diabetic milieu. Mechanistically, *Yap1* deletion was associated with disrupted MQC and mitochondrial aberrations within tubule cells, leading to discernible renal tubule injury and kidney impairments evidenced by histological examination. Strikingly, the administration of the YAP1 activator XMU-MP-1 showcased remarkable efficacy, counteracting these multifaceted events, even in the presence of persistent hyperglycemia and hyperlipidemia in diabetic mice. Our study also provides insights into potential therapeutic interventions. Overexpression of YAP1 via adenovirus-mediated gene transfer demonstrated the capacity to enhance mitochondrial membrane potential, attenuate mtROS production, sustain mtDNA copy numbers, mitophagy, OCR and the enzymatic activity of mitochondrial respiratory chain complexes, thereby maintaining MQC within cultured tubule cells exposed to PA and HG. These findings suggest that strategies aimed at restoring YAP1 activity possibly promote mitochondrial health within tubule cells, providing a mechanistic underpinning for the observed exacerbation of DKD in *Yap1* knockout mice.

Under the condition of HG or diabetes, the reduced efficiency of MQC has been confirmed by numerous scholars [14,15]. In agreement with prior numerous lines of evidence, PGC1α, a master regulator of mitobiogenesis was suppressed in both HK-2 cells treated with HG and STZ-induced diabetic kidney, which led to impaired mitobiogenesis and increased ROS generation [52,53]. Moreover, the PGC1α-centered mitochondrial dynamics disorder is often accompanied by changes in the expression of other mitobiogenesis-related mediators (TFAM and TOMM20) [54]. Additionally, in line with findings from our current study, previous evidence exists that renal tubular damage caused by disrupted mitophagy, selective macroautophagic targeting of damaged mitochondria, is closely related to DKD progression [3,12,13,55]. Kidney has a higher rate of mitophagy compared to other organs [56], and disruption of mitophagy may increase the need to use less favorable fuel sources such as glucose within tubule cells, leading to glucotoxicity in DKD [12,57]. The current mainstream view is that mitophagy has a renoprotective effect on DKD, while hyperglycemic-induced inhibition of PINK1-Parkin signaling in tubule cells will lead to incompetent mitophagy/autophagy flux, resulting in inflammatory and senescence responses [8,13,58,59].

Our study illuminated that *Yap1* deletion-induced renal tubule injury spurred the secretion of CXCL1 from tubule cells. This phenomenon was implicated in the augmented infiltration of macrophages into the renal microenvironment and the subsequent elevation in macrophage polarization [60]. Importantly, our study transcended the confines of experimental models, delving into the clinical realm. Bioinformatic analyses of transcriptomics data sourced from patients with diabetes corroborated the findings, revealing a significant upregulation of tubule cells-derived CXCL1 in tandem with infiltrating macrophages. This clinical validation substantiates the critical role played by tubule cells-derived chemokines in macrophage infiltration, emphasizing the translational significance of the study's findings [60,61]. In essence, this research not only expands our comprehension of the mechanisms underpinning DKD but also illuminates a promising avenue for therapeutic intervention, suggesting that the modulation of YAP1 and CXCL1 may hold promise as therapeutic strategies in human DKD and offering renewed hope for patients grappling with this debilitating condition [60].

## 5 Conclusion

**Fig. 8:**
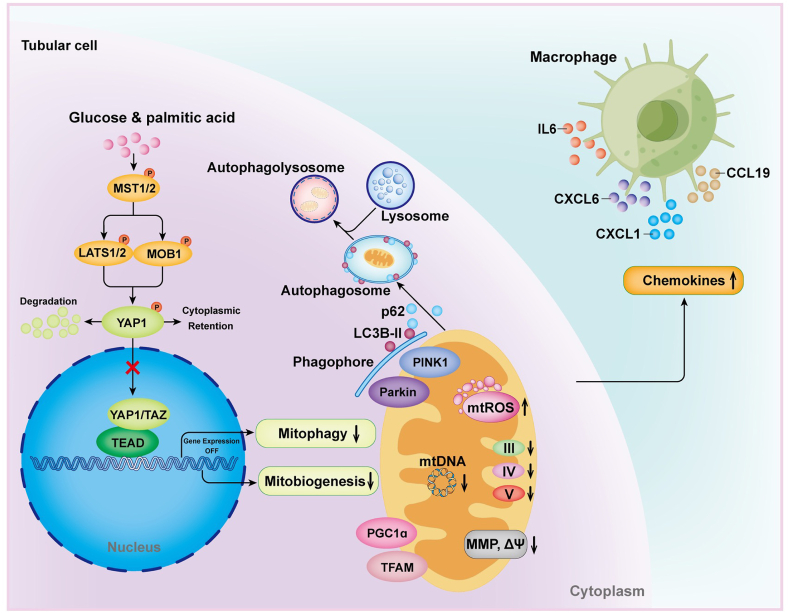
Schematic diagram showing the yes-associated protein 1 (YAP1) as a critical molecular pattern for diabetic kidney disease (DKD). The initiation of DKD within tubule cells triggers the activation of the Hippo signaling pathway, leading to the degradation of the downstream core mediator YAP1. This cascade results in the disruption of YAP1-mediated mitochondrial quality control, characterized by impaired mitobiogenesis and mitophagy. Consequently, there is an elevation in the production of mitochondrial reactive oxygen species (mtROS), a decline in mitochondrial membrane potential (MMP, ΔΨm), mtDNA copy numbers, oxygen consumption rate, and the enzymatic activity of mitochondrial respiratory chain complexes. These molecular alterations stimulate the synthesis of inflammatory cytokines (such as IL6) and chemokines (CXCL1, CXCL6, and CCL19) by tubule cells, instigating the recruitment and activation of macrophages. This intricate process significantly contributes to the pathogenesis of DKD. MST1, serine/threonine kinase 4; MST2, serine/threonine kinase 3; MOB1, MOB kinase activator 1; LATS1, large tumor suppressor kinase 1; LATS2, and large tumor suppressor kinase 2; TAZ, Tafazzin; TEAD, TEA domain transcription factor; PGC1ɑ, peroxisome proliferator-activated receptor γ coactivator α; TFAM, mitochondrial transcription factor A; PINK1, tensin homolog induced kinase 1; LC3B-Ⅱ, microtubule-associated protein 1 light chain 3B-Ⅱ; p62, ubiquitin-binding protein sequestosome 1; IL6, interleukin 6; CXCL1, C-X-C motif chemokine ligand 1; CCL19, C–C motif chemokine ligand; CXCL6, C-X-C motif chemokine ligand 6.

In summary, our study illuminates a multifaceted role for YAP1 in DKD, encompassing MQC regulation, immune response modulation, and potential therapeutic interventions. Renal tubule cell YAP1 inactivation-mediated MQC dysfunction emerges as a driving force in DKD progression, at least in part, by facilitating macrophage polarization via a paracrine-dependent mechanism (Fig. 8). The modulation of the Hippo signaling pathway, with a focus on restoring YAP1 activity, presents a promising avenue for preventing and halting the development and progression of DKD.

## CRediT authorship contribution statement

**Siyang Ye:** Writing – original draft, Methodology, Investigation, Formal analysis, Data curation, Conceptualization. **Meng Zhang:** Writing – original draft, Methodology, Investigation, Formal analysis, Data curation, Conceptualization. **Xunhua Zheng:** Validation, Methodology, Investigation, Formal analysis, Data curation. **Suchun Li:** Methodology, Investigation, Formal analysis, Data curation. **Yuting Fan:** Software, Methodology, Investigation, Data curation. **Yiqin Wang:** Validation, Methodology, Investigation, Formal analysis. **Huajing Peng:** Software, Methodology, Investigation, Data curation. **Sixiu Chen:** Validation, Methodology, Investigation. **Jiayi Yang:** Validation, Software, Methodology, Investigation. **Li Tan:** Validation, Methodology, Investigation. **Manhuai Zhang:** Software, Methodology, Investigation. **Peichen Xie:** Software, Methodology, Investigation. **Xiaoyan Li:** Validation, Methodology, Investigation. **Ning Luo:** Resources, Methodology, Investigation. **Zhipeng Wang:** Validation, Methodology, Investigation. **Leigang Jin:** Visualization, Methodology. **Xiaoping Wu:** Visualization, Software, Methodology. **Yong Pan:** Validation, Software. **Jinjin Fan:** Project administration, Methodology. **Yi Zhou:** Software, Project administration, Methodology. **Sydney C.W. Tang:** Writing – review & editing, Project administration, Methodology. **Bin Li:** Writing – original draft, Validation, Methodology, Investigation, Funding acquisition, Formal analysis, Data curation, Conceptualization. **Wei Chen:** Writing – review & editing, Project administration, Investigation.

## Data availability statement

The RNA-sequencing dataset has been deposited in the Gene Expression Omnibus database (accession number GSE256447). Microarray datasets were obtained from the GSE30529, GSE175759, and GSE104954, and single nucleus RNA-sequencing datasets were obtained from the GSE194560, GSE151302, and GSE131882. Any additional information to support the findings in the study is available from the corresponding authors on reasonable request.

## Funding

This work was supported by grants from the 10.13039/501100001809National Natural Science Foundation of China (Nos. 82170737, 82100747, and 82370707), 10.13039/501100003453Natural Science Foundation of Guangdong Province (No. 2024A1515013210), 10.13039/501100012166National Key Research and Development Project of China (No. 2021YFC2501302), 10.13039/501100018684NHC Key Laboratory of Clinical Nephrology (Sun Yat-Sen University), Guangdong Provincial Key Laboratory of Nephrology, Guangdong International Science and Technology Cooperation Institute of Immune Kidney Disease and Precision Medicine, General Project of 10.13039/501100003453Natural Science Foundation of Guangdong Province (No. 2019A1515010992), and Guangdong Medical Science and Technology Research Fund Project of China (No. A2020085). Part of the results from this study has been submitted for presentation in abstract form at the American Society of Nephrology Kidney Week 2022, Orlando, FL, USA, and ISN World Congress of Nephrology 2023, Bangkok, Thailand.

## Declaration of competing interest

The authors declare that they have no known competing financial interests or personal relationships that could have appeared to influence the work reported in this paper.

## Data Availability

Data will be made available on request.

## References

[1] Tuttle K.R., Agarwal R., Alpers C.E. (2022). Molecular mechanisms and therapeutic targets for diabetic kidney disease. Kidney Int..

[2] Zeni L., Norden A.G.W., Cancarini G. (2017). A more tubulocentric view of diabetic kidney disease. J. Nephrol..

[3] Yao L., Liang X., Liu Y. (2023). Non-steroidal mineralocorticoid receptor antagonist finerenone ameliorates mitochondrial dysfunction via PI3K/Akt/eNOS signaling pathway in diabetic tubulopathy. Redox Biol..

[4] Vallon V. (2011). The proximal tubule in the pathophysiology of the diabetic kidney. Am. J. Physiol. Regul. Integr. Comp. Physiol..

[5] Liu B.C., Tang T.T., Lv L.L. (2018). Renal tubule injury: a driving force toward chronic kidney disease. Kidney Int..

[6] Jia C., Ke-Hong C., Fei X. (2020). Decoy receptor 2 mediation of the senescent phenotype of tubular cells by interacting with peroxiredoxin 1 presents a novel mechanism of renal fibrosis in diabetic nephropathy. Kidney Int..

[7] Xu F., Jiang H., Li X. (2024). Discovery of PRDM16-mediated TRPA1 induction as the mechanism for low tubulo-interstitial fibrosis in diabetic kidney disease. Adv. Sci..

[8] Han Y., Xu X., Tang C. (2018). Reactive oxygen species promote tubular injury in diabetic nephropathy: the role of the mitochondrial ros-txnip-nlrp3 biological axis. Redox Biol..

[9] Doke T., Susztak K. (2022). The multifaceted role of kidney tubule mitochondrial dysfunction in kidney disease development. Trends Cell Biol..

[10] Li H., Leung J.C.K., Yiu W.H. (2022). Tubular beta-catenin alleviates mitochondrial dysfunction and cell death in acute kidney injury. Cell Death Dis..

[11] Tang C., Cai J., Yin X.M. (2021). Mitochondrial quality control in kidney injury and repair. Nat. Rev. Nephrol..

[12] Liu L., Bai F., Song H. (2022). Upregulation of TIPE1 in tubular epithelial cell aggravates diabetic nephropathy by disrupting PHB2 mediated mitophagy. Redox Biol..

[13] Xiao L., Xu X., Zhang F. (2017). The mitochondria-targeted antioxidant MitoQ ameliorated tubular injury mediated by mitophagy in diabetic kidney disease via Nrf2/PINK1. Redox Biol..

[14] Chung K.W., Dhillon P., Huang S. (2019). Mitochondrial damage and activation of the STING pathway lead to renal inflammation and fibrosis. Cell Metabol..

[15] Galvan D.L., Green N.H., Danesh F.R. (2017). The hallmarks of mitochondrial dysfunction in chronic kidney disease. Kidney Int..

[16] Fu J., Sun Z., Wang X. (2022). The single-cell landscape of kidney immune cells reveals transcriptional heterogeneity in early diabetic kidney disease. Kidney Int..

[17] Haruhara K., Suzuki T., Wakui H. (2022). Deficiency of the kidney tubular angiotensin II type1 receptor-associated protein ATRAP exacerbates streptozotocin-induced diabetic glomerular injury via reducing protective macrophage polarization. Kidney Int..

[18] Zhang M.Z., Wang X., Wang Y. (2017). IL-4/IL-13-mediated polarization of renal macrophages/dendritic cells to an M2a phenotype is essential for recovery from acute kidney injury. Kidney Int..

[19] Anders H.J., Vielhauer V., Schlondorff D. (2003). Chemokines and chemokine receptors are involved in the resolution or progression of renal disease. Kidney Int..

[20] Eardley K.S., Zehnder D., Quinkler M. (2006). The relationship between albuminuria, MCP-1/CCL2, and interstitial macrophages in chronic kidney disease. Kidney Int..

[21] Tesch G.H. (2008). MCP-1/CCL2: a new diagnostic marker and therapeutic target for progressive renal injury in diabetic nephropathy. Am. J. Physiol. Ren. Physiol..

[22] Hu Y., Tang W., Liu W. (2022). Astragaloside IV alleviates renal tubular epithelial-mesenchymal transition via cx3cl1-RAF/MEK/ERK signaling pathway in diabetic kidney disease. Drug Des. Dev. Ther..

[23] Chang T.T., Chen J.W. (2020). The role of chemokines and chemokine receptors in diabetic nephropathy. Int. J. Mol. Sci..

[24] Fu M., Hu Y., Lan T. (2022). The Hippo signalling pathway and its implications in human health and diseases. Signal Transduct. Targeted Ther..

[25] Guerin A., Angebault C., Kinet S. (2022). LIX1-mediated changes in mitochondrial metabolism control the fate of digestive mesenchyme-derived cells. Redox Biol..

[26] Chen J., Wang X., He Q. (2020). YAP activation in renal proximal tubule cells drives diabetic renal interstitial fibrogenesis. Diabetes.

[27] Zhang Y., Huang H., Kong Y. (2023). Kidney tubular transcription co-activator, Yes-associated protein 1 (YAP), controls the expression of collecting duct aquaporins and water homeostasis. Kidney Int..

[28] Choi S., Hong S.P., Bae J.H. (2023). Hyperactivation of YAP/TAZ drives alterations in mesangial cells through stabilization of N-myc in diabetic nephropathy. J. Am. Soc. Nephrol..

[29] Wong J.S., Meliambro K., Ray J. (2016). Hippo signaling in the kidney: the good and the bad. Am. J. Physiol. Ren. Physiol..

[30] Jiang M., Wei Q., Dong G. (2012). Autophagy in proximal tubules protects against acute kidney injury. Kidney Int..

[31] Mathers K.E., Staples J.F. (2019). Differential posttranslational modification of mitochondrial enzymes corresponds with metabolic suppression during hibernation. Am. J. Physiol. Regul. Integr. Comp. Physiol..

[32] Peng Y., Liu H., Liu J. (2022). Post-translational modifications on mitochondrial metabolic enzymes in cancer. Free Radic. Biol. Med..

[33] Finley L.W., Haas W., Desquiret-Dumas V. (2011). Succinate dehydrogenase is a direct target of sirtuin 3 deacetylase activity. PLoS One.

[34] Lkhagva B., Kao Y.H., Lee T.I. (2018). Activation of Class I histone deacetylases contributes to mitochondrial dysfunction in cardiomyocytes with altered complex activities. Epigenetics.

[35] Bezawork-Geleta A., Rohlena J., Dong L. (2017). Mitochondrial complex II: at the crossroads. Trends Biochem. Sci..

[36] Vercellino I., Sazanov L.A. (2022). The assembly, regulation and function of the mitochondrial respiratory chain. Nat. Rev. Mol. Cell Biol..

[37] Sharma L.K., Lu J., Bai Y. (2009). Mitochondrial respiratory complex I: structure, function and implication in human diseases. Curr. Med. Chem..

[38] Sanchez-Caballero L., Guerrero-Castillo S., Nijtmans L. (2016). Unraveling the complexity of mitochondrial complex I assembly: a dynamic process. Biochim. Biophys. Acta.

[39] Stroud D.A., Surgenor E.E., Formosa L.E. (2016). Accessory subunits are integral for assembly and function of human mitochondrial complex I. Nature.

[40] Ma R., Ren J.M., Li P. (2019). Activated YAP causes renal damage of type 2 diabetic nephropathy. Eur. Rev. Med. Pharmacol. Sci..

[41] Wang Y., Xu J., Cheng Z. (2021). YAP1 promotes high glucose-induced inflammation and extracellular matrix deposition in glomerular mesangial cells by modulating NF-kappaB/JMJD3 pathway. Exp. Ther. Med..

[42] Wan H., Wang Y., Pan Q. (2022). Quercetin attenuates the proliferation, inflammation, and oxidative stress of high glucose-induced human mesangial cells by regulating the miR-485-5p/YAP1 pathway. Int. J. Immunopathol. Pharmacol..

[43] Bonse J., Wennmann D.O., Kremerskothen J. (2018). Nuclear YAP localization as a key regulator of podocyte function. Cell Death Dis..

[44] Schwartzman M., Reginensi A., Wong J.S. (2016). Podocyte-specific deletion of yes-associated protein causes FSGS and progressive renal failure. J. Am. Soc. Nephrol..

[45] Meliambro K., Wong J.S., Ray J. (2017). The Hippo pathway regulator KIBRA promotes podocyte injury by inhibiting YAP signaling and disrupting actin cytoskeletal dynamics. J. Biol. Chem..

[46] Zhuang Q., Li F., Liu J. (2021). Nuclear exclusion of YAP exacerbates podocyte apoptosis and disease progression in Adriamycin-induced focal segmental glomerulosclerosis. Lab. Invest..

[47] Rinschen M.M., Grahammer F., Hoppe A.K. (2017). YAP-mediated mechanotransduction determines the podocyte's response to damage. Sci. Signal..

[48] Chen J., Harris R.C. (2016). Interaction of the EGF receptor and the Hippo pathway in the diabetic kidney. J. Am. Soc. Nephrol..

[49] Huang Z., Peng Y., Ke G. (2023). CaMKII may regulate renal tubular epithelial cell apoptosis through YAP/NFAT2 in acute kidney injury mice. Ren. Fail..

[50] Chen J., You H., Li Y. (2018). EGF receptor-dependent YAP activation is important for renal recovery from AKI. J. Am. Soc. Nephrol..

[51] Xu J., Li P.X., Wu J. (2016). Involvement of the Hippo pathway in regeneration and fibrogenesis after ischaemic acute kidney injury: YAP is the key effector. Clinical science (London, England : 1979).

[52] Broome S.C., Pham T., Braakhuis A.J. (2022). MitoQ supplementation augments acute exercise-induced increases in muscle PGC1alpha mRNA and improves training-induced increases in peak power independent of mitochondrial content and function in untrained middle-aged men. Redox Biol..

[53] Sekar P., Hsiao G., Hsu S.H. (2023). Metformin inhibits methylglyoxal-induced retinal pigment epithelial cell death and retinopathy via AMPK-dependent mechanisms: reversing mitochondrial dysfunction and upregulating glyoxalase 1. Redox Biol..

[54] Ma H., Guo X., Cui S. (2022). Dephosphorylation of AMP-activated protein kinase exacerbates ischemia/reperfusion-induced acute kidney injury via mitochondrial dysfunction. Kidney Int..

[55] Liu Y., Chen W., Li C. (2023). DsbA-L interacting with catalase in peroxisome improves tubular oxidative damage in diabetic nephropathy. Redox Biol..

[56] Sun N., Yun J., Liu J. (2015). Measuring in vivo mitophagy. Mol. Cell.

[57] Sakai S., Yamamoto T., Takabatake Y. (2019). Proximal tubule autophagy differs in type 1 and 2 diabetes. J. Am. Soc. Nephrol..

[58] Forbes J.M., Thorburn D.R. (2018). Mitochondrial dysfunction in diabetic kidney disease. Nat. Rev. Nephrol..

[59] Chen K., Feng L., Hu W. (2019). Optineurin inhibits NLRP3 inflammasome activation by enhancing mitophagy of renal tubular cells in diabetic nephropathy. Faseb. J..

[60] Wang S., Bai J., Zhang Y.L. (2022). CXCL1-CXCR2 signalling mediates hypertensive retinopathy by inducing macrophage infiltration. Redox Biol..

[61] Zhao S., Zhou L., Wang Q. (2023). Elevated branched-chain amino acid promotes atherosclerosis progression by enhancing mitochondrial-to-nuclear H(2)O(2)-disulfide HMGB1 in macrophages. Redox Biol..

