# Supplementary material for: YAP1 preserves tubular mitochondrial quality control to mitigate diabetic kidney disease
Source: Redox Biol. 2024 Nov 23;78:103435. doi: 10.1016/j.redox.2024.103435 (PMC11629574; doi:10.1016/j.redox.2024.103435)
Supplement: Multimedia component 1 [file mmc1.docx]

**Supplementary data**

**YAP1 Preserves Tubular Mitochondrial Quality Control to Mitigate Diabetic Kidney Disease**

Siyang Ye^1^, Meng Zhang^1^, Xunhua Zheng^1^, Suchun Li, Yuting Fan, Yiqin Wang, Huajing Peng, Sixiu Chen, Jiayi Yang, Li Tan, Manhuai Zhang, Peichen Xie, Xiaoyan Li, Ning Luo, Zhipeng Wang, Leigang Jin, Xiaoping Wu, Yong Pan, Jinjin Fan, Yi Zhou, Sydney C.W. Tang^*^, Bin Li^**^ and Wei Chen^***^

**Materials and methods**

**1. Human subjects**

Renal biopsy samples from DKD patients and control donors were obtained from the First Affiliated Hospital, Sun Yat-sen University, Guangzhou, China. The control samples were para-carcinoma tissues from individuals who underwent tumor nephrectomies without other kidney diseases. DKD patients were divided into two groups: ‘early-stage DKD’ and ‘late-stage DKD’. The early-stage DKD group was defined as patients with diabetes who had Stage 1, 2 or 3a chronic kidney disease (CKD), while the late-stage DKD group was defined as patients with diabetes who had Stage 3b, 4 or 5 CKD^1^. This study was conducted following the principles of the Declaration of Helsinki and approved by the Ethics Committee of the First Affiliated Hospital, Sun Yat-sen University (Approval number [2016] 215) after informed consent was obtained from all patients.

**2. Generation of tubule cell-specific *Yap1* knockout mice**

Renal tubule cell-specific *Yap1* knockout mice (TKO, *KspCre*/*Yap1^flox/flox^*) and their control counterparts lacking the *KspCre* allele (CTL, *WT/Yap1^flox/flox^*) were bred at the Shanghai Model Organisms Center in China. The generation of TKO mice involved the mating of *Yap1^flox/flox^* mice (C57BL/6Smoc-Yap1^tm1(flox)Smoc^, Cat. No. NM-CKO-200174, Shanghai Model Organisms Center, Inc.) with *KspCre* transgenic mice (B6.Cg-Tg(Cdh16-Cre)91Igr/J*,* RRID: IMSR_JAX: 012237, Jackson Laboratory) that expressed Cre recombinase under the control of kidney-specific-cadherin promoter. All mice were genotyped by polymerase chain reaction (PCR) with specific primers (**Supplementary Table S1**) using tail-snip DNA. Only age-matched male mice were used for the subsequent experiments. Following two weeks of quarantine, mice were housed in a specific pathogen-free environment in the Laboratory Animal Center of Sun Yat-sen University, with a 12-hour light/dark cycle.

**3. High-fat diet (HFD) and streptozotocin (STZ)-induced DKD in mice**

The mice were categorized into four groups: (1) CTL mice on a normal diet (CTL+Veh+ND) group (n=6); (2) TKO mice on a normal diet (TKO+Veh+ND) group (n=6); (3) CTL mice on a high-fat diet (HFD) with streptozotocin (STZ) administration (CTL+STZ+HFD) group (n=6); and (4) TKO mice on a HFD with STZ administration (TKO+STZ+HFD) group (n=6). Mice in the ND or HFD groups were fed either a standard chow diet (containing 10% fat, 20% protein, and 70% carbohydrates; Research Diets, New Brunswick, NJ, USA) or a HFD (comprising 60% fat, 20% protein, and 20% carbohydrates; Research Diets) continuously for 22 weeks. After eight weeks on the HFD, mice in the STZ+HFD groups were intraperitoneally injected with STZ (40 mg/kg; Sigma‐Aldrich, St. Louis, MO, USA) dissolved in ice-cold sodium citrate (pH 4.5; Phygene, Fuzhou, Fujian, China) for five consecutive days, while the Veh+ND groups received the sodium citrate vehicle (**Figure 3A**). Blood glucose levels were measured two weeks post-STZ injection, and mice with blood glucose levels exceeding 13.9 mmol/L were considered as diabetic mice.

**4. Administration of YAP1 activator XMU-MP-1 in mice**

Additionally, XMU-MP-1 (Selleck, Shanghai, China), dissolved in solvent (2% DMSO [MP Biomedicals, Solon, OH, USA], 30% PEG300 [Selleck], 2% Tween80 [Selleck], and ddH_2_O), was administered intraperitoneally at a dosage of 1 mg/kg body weight every two days to wild-type diabetic mice induced by a combination of HFD and STZ intraperitoneal injection^2-4^. The solvent of XMU-MP-1 was used as vehicle control in this experiment. The dosing regimen was based on the pharmacokinetic data of XMU-MP-1 in a rodent model^3^, as well as functional assays demonstrating the efficacy of XMU-MP-1 injection (1 mg/kg) in reducing MOB1 phosphorylation in mice^3, 4^. XMU-MP-1 treatment commenced two weeks post intraperitoneal STZ injection and continued for an additional 12 weeks (**Figure 6A**). At the conclusion of the experiments, mice were anesthetized and humanely euthanized by cervical dislocation, and mice samples were collected for further analysis. The animal experimental protocols adhered to ethical guidelines and were approved by the Institutional Animal Care and Use Committee of Sun Yat-sen University in China (Approval number SYSU-IACUC-2021-000966, SYSU-IACUC-2022-001525).

**5. Blood and urine analyses**

Blood glucose levels were measured by tail vein blood sampling using ACCU-CHEK Performa blood glucose analyzer (Roche Diagnostics, Mannheim, Germany). Circulating blood triglyceride, cholesterol, low-density lipoprotein cholesterol (LDL-c), and creatinine levels were measured in serum samples using commercial assay kits (Roche Diagnostics) on a COBAS C311 automatic biochemistry analyzer (Roche Diagnostics). Urinary albumin and creatinine levels were measured using commercial assay kits (BIOBASE, Jinan, Shandong, China) on a BK-280 automatic biochemistry analyzer (BIOBASE).

**6. Renal histology and immunohistochemistry**

Renal tissue specimens were fixed with 4% paraformaldehyde and embedded in paraffin. Subsequently, paraffin‐embedded kidney sections (4 µm) were subjected to Periodic acid-Schiff (PAS) staining, Masson trichrome staining, and immunohistochemistry. PAS staining was performed using a PAS staining Kit (Biossci Biotechnology, Wuhan, Hubei, China) according to the manufacturer’s instructions. Masson trichrome staining was conducted using a Trichrome staining (Masson) Kit (Biossci Biotechnology) in accordance to the manufacturer’s suggested protocol. Tubular injury in renal cortex was determined and graded using 10 random nonoverlapping fields from each sample at ×400 magnification and assessment of four categories of damage was made including presence of tubular necrosis, loss of brush border, cast formation and tubular dilatation^5^. The injury score was calculated as the sum of this semiquantitative assessment of tubular injury. The relative collagen area of the tubulointerstitial region was observed and quantified by ImageJ Software in 20 randomly selected cortical glomeruli from each sample at ×400 magnification and analyzed in a blinded fashion. For immunohistochemistry, the sections were incubated overnight at 4℃ with primary antibody. After rinsing in phosphate buffer saline (PBS), slides were exposed for 1 hour to the secondary antibody (Agilent Dako, Santa Clara, CA, USA). The sections were later washed with PBS, followed by incubation with diaminobenzidine (DAB) for detection. Microscopic images were captured at low and high magnification using the ZEISS Axio Imager Z2 light microscope system (Carl Zeiss, Gottingen, Germany). Quantitative analysis of the immunohistochemistry staining in randomly selected 20 fields in the cortex from each sample was measured by ImageJ software to evaluate percentage of positive cell optical density relative to total area in each field under the same light intensity as for microscopy. Detailed information about the antibodies used was provided in the **Supplementary Table S2**.

**7. Quantitative real-time PCR**

The total RNA was extracted with Trizol (Invitrogen, Carlsbad, CA, USA) and converted into cDNA using the Transcriptor First Strand cDNA Synthesis Kit (Roche Diagnostics) according to the manufacturer’s protocol. Quantitative real-time polymerase chain reaction (qRT-PCR) analyses were conducted on a QuantStudio5 Real-Time PCR System (Applied Biosystems, Foster City, CA, USA) employing FastStart Universal SYBR Green Master Mix (Roche Diagnostics) and specific primers. Relative mRNA levels of genes were normalized to those of glyceraldehyde 3-phosphate dehydrogenase (GAPDH). The primer sequences were listed in the **Supplementary Table S1**.

**8. Western blot analyses**

Cells or renal cortical tissues were lysed with RIPA buffer (1% NP40, 0.1% SDS, 0.5% sodium deoxycholate, 5 mM EDTA, 1X PBS) containing both protease and phosphatase inhibitor cocktails (Roche Diagnostics). The nuclear and cytoplasmic fractions were extracted following the manual of the Nuclear and Cytoplasmic Protein Extraction Kit (Beyotime Biotechnology, Shanghai, China). The protein concentrations were quantified using the Pierce BCA Protein Assay Kit (Thermo Scientific, Waltham, MA, USA). Equal amounts of protein were separated on 4%-12% SurePAGE Bis-Tris precast gels (GenScript, Nanjing, Jiangsu, China), and subsequently transferred onto 0.2 µm polyvinylidene fluoride membranes (Merck-Millipore, Darmstadt, Germany) through standard electroblotting procedures. The membranes were then blocked with 5% skim milk in Tris-buffered saline containing 0.1% Tween20 (TBST) for 30 min at room temperature. Following this, the membranes were thoroughly washed with TBST, incubated with primary antibodies overnight at 4℃, washed with TBST, and incubated with appropriate secondary antibodies for 1 hour at room temperature. Subsequently, the membranes were washed with TBST before detecting protein bands using Immobilon Western Chemiluminescent HRP Substrate (Merck-Millipore). Western blots were imaged using the ChemiScope 6200 Touch Chemiluminescence Imaging System (Clinx Science Instruments, Shanghai, China). Detailed information about the antibodies used was provided in the **Supplementary Table S2**.

**9. Cell culture and treatment**

The human renal proximal tubular cells (HK-2), obtained from the American Type Culture Collection (ATCC), were cultured in Dulbecco’s Modified Eagle’s Medium (DMEM)/F12 (Gibco, Carlsbad, CA, USA) supplemented with 10% FBS (Gibco) and 1% penicillin/streptomycin (Gibco) at 37 °C under humidified air containing 5% CO_2_. Prior to conducting experiments, cells were synchronized into quiescence by serum starvation for 12 hours. Given that diabetic individuals or mice commonly exhibit hyperglycemia, hyperlipidemia, and increased levels of circulating free fatty acids, we postulated that these factors might contribute to renal injury in this context. To simulate these conditions, we exposed HK-2 cells to high glucose (HG, 40 mM; ELGBIO Biotechnology, Guangzhou, Guangdong, China), palmitic acid (PA, 500 μM; Sigma‐Aldrich, St. Louis, MO, USA), or a combination of both for 24 hours. PA was specifically chosen due to its status as a major long-chain saturated fatty acid recognized for its ability to disrupt cellular metabolism, elevate oxidative stress, and induce inflammation^6^. For overexpression of YAP1 in HK-2 cells, adenovirus encoding the human *YAP1* gene (Ad-YAP1) and negative control adenovirus (Ad-NC) were constructed and produced by OBiO Technology Corp., Ltd (Shanghai, China). Briefly, the entire coding region of the human *YAP1* gene was introduced to a replication-defective adenoviral vector under the control of the cytomegalovirus promoter. Recombinant adenoviruses expressing Flag-tagged YAP1 protein were purified. HK-2 cells were infected with the adenovirus at a multiplicity of infection of 100. To inhibit mitophagy, HK-2 cells were pretreated with 5 μM Mdivi-1 (Selleck) for 6 hours before exposure to PA and HG.

**10. Immunofluorescence staining**

Kidney sections or cells cultured on sterile glass coverslips were prepared for immunofluorescence staining according to standard techniques. Paraffin‐embedded kidney sections were dewaxed, rehydrated, and autoclaved for 10 min in EDTA buffer (pH 9.0) for antigen retrieval. Cryosections and cultured cells were fixed with 4% paraformaldehyde for 30 min at room temperature. After being blocked with 10% goat/donkey serum or 5% BSA containing 0.5% Triton X-100 for 30 min at room temperature, the kidney sections and cell coverslips were incubated with primary antibodies at 4℃ overnight. After washing with PBS, the sections and coverslips were incubated with appropriate secondary antibodies at room temperature for 1 hour in the dark. Finally, blue-fluorescent DAPI was used to counterstain the nuclei. Images were acquired using the ZEISS Axio Imager Z2 light microscope system (Carl Zeiss). Detailed information about the antibodies used was provided in the **Supplementary Table S2**.

**11. Transmission electron microscopy**

Cells or renal cortical tissue blocks (1 mm^3^) were fixed with 2.5% glutaraldehyde at 4˚C overnight. The prefixed samples were washed with PBS and postfixed with 1% osmium tetroxide for 2 hours at room temperature. The samples were then washed again, dehydrated through an ascending alcohol gradient, and finally embedded. Ultrathin sections (100 nm) were cut with a Leica EM UC7 ultramicrotome (Leica Microsystems, Wetzlar, Germany), stained with uranyl acetate (20 min) and lead citrate (12 min), and were viewed with a Tecnai G2 Spirit transmission electron microscope (FEI, Hillsboro, OR, USA).

**12. Mitochondrial membrane potential**

The mitochondrial membrane potential (MMP) of cells was determined using a commercial MMP Assay Kit with JC-1 (Beyotime Biotechnology, Shanghai, China). Briefly, the harvested cells were resuspended in a mixture containing 500 μL of culture medium and 500 μL of JC-1 (5 μg/mL) staining working solution. Subsequently, the cells were incubated at 37 °C under humidified air containing 5% CO_2_ for 20 min in the dark. After the cells were washed twice with ice-cold sterile PBS through centrifugation, the cell clumps were resuspended in 500 μl of PBS for further analysis. JC-1 fluorescence of cells was measured by Attune NxT flow cytometer (Thermo Fisher, Waltham, MA, USA), in which red JC-1 aggregates were gated in the YL-1 channel (excited by 561 nm laser) and green JC-1 monomers in the BL-1 channel (excited by 488 nm laser). The red fluorescence (aggregated JC-1)/green fluorescence (monomeric JC-1) ratio was calculated to represent MMP^7^.

**13. Mitochondrial ROS measurement**

Mitochondrial ROS levels were measured by MitoSox Red mitochondrial superoxide indicator (Invitrogen, Carlsbad, CA, USA). Briefly, 50 μg MitoSox Red was dissolved in 13 μL DMSO (MP Biomedicals, Solon, OH, USA) to make 5 mM MitoSox stock solution. Then, the stock solution was diluted 1,000-fold in Hankʼs balanced salt solution (HBSS) to make a 5 μM MitoSox working solution. Cells were exposed to 1 mL MitoSox working solution and then incubated at 37 °C under humidified air containing 5% CO_2_ for 10 min, protected from light. Cells were collected and washed three times by warm HBSS, and the mitochondrial ROS levels of cells were analyzed by flow cytometry. The mean fluorescence intensity (MFI) for each sample was calculated using FlowJo v10.8.1 Software (BD Life Sciences, Ashland, OR, USA).

**14. Live mt-Keima imaging**

HK2 cells were transfected with adenoviruses encoding mitochondria-targeted mt-Keima (Hanbio Biotechnology, Shanghai, China) at the indicated multiplicity of infection according to the manufacturer’s instructions^8^. The pH-dependent mt-Keima was detected as a red fluorescent signal (excited by 561 nm laser) at acidic pH (lysosome) and a green fluorescent signal (excited by 488 nm laser) at neutral pH (mitochondria) by a Zeiss LSM 900 confocal microscope (Carl Zeiss). The acquisition parameters were kept constant for all images during confocal microscopy. To measure mitophagy in individual cells, the mt-Keima fluorescent images were captured and analyzed in a blinded fashion. Approximately 10 cells were randomly captured and analyzed per condition per experiment. The mitophagy index was determined by the ratio of red (mt-Keima in acidic pH): green (mt-Keima in neutral pH) fluorescence^8, 9^.

**15. Oxygen consumption rate (OCR) measurements**

OCR was measured using the Seahorse Extracellular Flux (XFe96) analyzer (Agilent Technologies, Santa Clara, CA, USA) and the Seahorse XF Cell Mito Stress Test Kit (Agilent Technologies). Briefly, the indicated HK-2 cells were seeded at 20,000 cells per well (80 μL volume) of a Seahorse XF96 cell culture plate and incubated at 37 °C under humidified air containing 5% CO_2_ overnight. After incubation overnight, cells were washed with freshly prepared XF Assay Medium (Seahorse XF DMEM medium [Agilent Technologies] supplemented with 10 mM glucose [Agilent Technologies], 1 mM pyruvate [Agilent Technologies], 2 mM L-glutamine [Agilent Technologies]), and then cells were kept in 180 μL per well of XF Assay Medium at 37 °C incubator without CO_2_ for 1 hour prior to the measurement. OCR was measured before and after sequential injection of Oligomycin (1.5 µM), FCCP (1 µM) and Rotenone (0.5 µM) plus Antimycin A (0.5 µM). Agilent Seahorse Wave Desktop v2.6.3 Software (Agilent Technologies) was used for report generation and data analysis.

**16. Detection of mitochondrial respiratory chain complex activity**

Mitochondrial respiratory chain complex activity assay kits (Abbkine, Wuhan, China) were used to detect the activities of mitochondrial respiratory chain complex I-V in accordance with the manufacturer’s instructions. The absorption values at 340 nm (complex I), 605 nm (complex II), 550 nm (complexes III and IV), and 660 nm (complex V) were measured using a SpectraMax 190 microplate reader (Molecular Devices, San Jose, CA, USA). The activities of mitochondrial respiratory chain complexes were calculated based on the calculation formulae provided by the manufacturer.

**17. Measurement of mitochondrial DNA (mtDNA) copy number**

Total genomic DNA was isolated using the SpeeDNA Isolation Kit (ScienCell Research Laboratories, San Diego, CA, USA) and mtDNA copy number was measured using the Relative Human mtDNA Copy Number Quantification qPCR Assay Kit (ScienCell Research Laboratories) according to the manufacturer’s protocol. All primers (mtDNA primer set and single copy reference primer set) were supplied in the above mtDNA Copy Number Quantification qPCR Assay Kit. The mtDNA primer set recognizes and amplifies one of the most conserved regions on human mtDNA. The single copy reference primer set recognizes and amplifies a 100 bp-long region on human chromosome 17 and serves as reference for data normalization.

**18. Measurement of CXCL1 secreted in the culture medium**

Cell culture media from HK-2 cells were harvested and centrifuged after the indicated treatments. CXCL1 levels in the culture media were determined by enzyme-linked immunosorbent assay (ELISA) with a Human CXCL1 Double-antibody Sandwich ELISA Kit (ELGBIO Biotechnology). Assays were performed in accordance with the manufacturer’s instructions. The optical density values at 450 nm were measured using a SpectraMax 190 microplate reader (Molecular Devices). The CXCL1 levels in the culture media were calculated based on the standard curve.

**19. Retrieval of microarray dataset collection, identification of differentially expressed genes (DEGs) and Gene Ontology analyses**

The high-throughput gene expression datasets, GSE30529 (N=12 control, N=10 DKD)^10^, GSE175759 (N=22 control, N=3 DKD)^11^, and GSE104954 (N=21 control, N=17 DKD)^12^, were sourced from the Gene Expression Omnibus (GEO) and underwent initial standardization and normalization using Transcripts Per Kilobase Million (TPM)^13^. Differentially expressed genes (DEGs) were identified utilizing the R edgeR package^14^. Genes with a ∣log2foldchange∣ > 0.58 and a *P* value < 0.05 were categorized as DEGs between control and DKD samples. Volcano plots and heatmap clusters depicting the DEGs were generated using the ggplot modules within the R package. The overlapping DEGs across all three sample groups were meticulously scrutinized to pinpoint the commonly shared DEGs crucial in the pathogenesis of DKD. Subsequently, these commonly shared DEGs underwent Gene Ontology (GO) and Kyoto Encyclopedia of Genes and Genomes (KEGG) pathway enrichment analysis, facilitated by the clusterprofiler package. For in-depth details regarding the datasets, encompassing the intricate procedures of human RNA extraction from tubulointerstitial compartments, sample preparation, microarray processing, and gene-expression data analysis, readers are directed to the respective original literature and **Supplementary Table S3**.

**20. Retrieval of single nucleus RNA sequencing dataset collection**

The sing nucleus RNA sequencing (snRNA-seq) data was derived from the Gene Expression Omnibus (GEO) dataset with the accession number GSE195460 (N=1 control, N=2 DKD)^15^, GSE151302 (N=5 control)^16, 17^, and GSE131882 (N=3 control, N=3 DKD)^18^. The analysis of snRNA-seq data was conducted by the R package Seurat^19^, and data filtering and normalization were carried out. Moreover, unsupervised clustering was performed via uniform manifold approximation and projection (UMAP) and t-distributed stochastic neighbor embedding (t-SNE), while the immune cell cluster was manually annotated using the known immune cell marker genes (CD163, CSF1R, CD247, CD96, MS4A1, PAX5, CD38, SDC1). Each cluster was assigned to the compartment of its maximal score and all cluster assignments were manually checked to ensure the accurate partition of cells. We used the “FindAllMarkers” function in Seurat to identify genes that are differentially expressed between clusters with the following parameters: min.pct=0.1, logfc.threshold=0.25, pseudocount.use=0.1, only.pos=T. The non-parametric Wilcoxon rank-sum test was used to obtain *P*-values for comparisons, and the adjusted *P*-values, based on Bonferroni correction, for all genes in the dataset. Bubble charts were used to visualize *CXCL1*, *CCL19*, and *CXCL6* based on gene expression after the log-transformed and scaling. Additional information about all the above three datasets, including the detailed procedure of human snRNA extraction from the glomeruli and tubulointerstitial compartments, sample preparation and microarray processing, as well as gene-expression data analysis could be acquired from the corresponding original literature and **Supplementary Table S4**.

**21. RNA-sequencing in mice kidney and GSEA bioinformatic analysis**

Kidney tissues of (XMU-MP-1)-treated diabetic mice and the controls were collected for RNA sequencing (N=3 in each group). Total RNA from kidneys was isolated by using Trizol in accordance with the manufacturer’s instructions and then followed by an additional DNase I digestion to remove genomic DNA contamination. RNA quality and purity was checked by using the NanoDrop ND-1000 spectrophotometer (NanoDrop Technologies, Wilmington, DE, USA). And then total RNA samples were used to remove highly abundant ribosomal RNAs before sequencing. The sequencing was done by Illumina Genome Analyzer (serial number Hiseq2000) as previously reported^20^. Both library building and sequencing were performed by LC-Bio Technology Co., Ltd (Hangzhou, Zhejiang, China). Low-quality reads, over 50% of the sequences reads (including adapters) has quality score ≤10 and N > 5%, were discarded. Ribosome RNA sequences were filtered from the raw fragments. Those left clean reads were mapped to genome (UCSC genome browser, Version: July.2007, mm9) by using Tophat [1.3.0.Linux_x86_64 (bowtie-0.12.7)] and then assembled with Cufflinks (1.3.0.Linux_x86_64)^21^. Gene set enrichment analysis (GSEA) was performed with Broad GSEA software (version 4.0.2) using the hallmark gene sets (h.all.v.7.4.symbols.gmt) in MSigDB for pathway annotation. The sequencing data obtained were available at the Gene Expression Omnibus Web site (<http://www.ncbi.nlm.nih.gov/geo/>, accession number GSE256447).

**22. Evaluation of immune cell infiltration by CIBERSORT analyses**

We utilized the CIBERSORT analytical platform (<https://cibersort.stanford.edu/>) with 1,000 permutations and the LM22 signature to comprehensively characterize the immune cell landscape within kidney tissues. CIBERSORT employs a validated deconvolution algorithm, offering a robust and precise method for estimating immune cell fractions from bulk transcriptome data. This method allows the identification and quantification of 22 distinct immune cell types, including but not limited to naive B cells, memory B cells, plasma cells, CD8+ T cells, naive CD4+ T cells, resting memory CD4+ T cells, activated memory CD4+ T cells, follicular helper T cells, regulatory T cells (Tregs), gamma delta T cells, resting natural killer (NK) cells, activated NK cells, monocytes, M0 macrophages, M1 macrophages, M2 macrophages, resting dendritic cells, activated dendritic cells, resting mast cells, activated mast cells, eosinophils, and neutrophils. Significant alterations in immune cell composition were identified using the Wilcoxon test, with a threshold *p*-value of less than 0.05.

**Table S1. Primer sequence of target genes used in this study.**

| **Gene Name** | **Species** | **Forward 5'-3'** | **Reverse 5'-3'** | **Application** |
| --- | --- | --- | --- | --- |
| *Yap1-flox* | Mouse | CCGTTTCTCCTGGGACACTC | CCACAAAAGTCTGCAAAAAGGC | PCR (Genotyping) |
| *Cdh16-Cre* | Mouse | GCAGATCTGGCTCTCCAAAG | AGGCAAATTTTGGTGTACGG | PCR (Genotyping) |
| *YAP1* | Human | TGACCCTCGTTTTGCCATGA | GTTGCTGCTGGTTGGAGTTG | Quantitative real-time PCR |
| *PGC1α* | Human | AAAGGATGCGCTCTCGTTCA | CTTCAGCCTCTCGTGCTGAT | Quantitative real-time PCR |
| *TFAM* | Human | GGCAAGTTGTCCAAAGAAACC | GCATCTGGGTTCTGAGCTTTA | Quantitative real-time PCR |
| *TOMM20* | Human | GGTACTGCATCTACTTCGACCG | TGGTCTACGCCCTTCTCATATTC | Quantitative real-time PCR |
| *PINK1* | Human | GTGGAACATCTCGGCAGGTT | CCTCTCTTGGATTTTCTGTAAGTGAC | Quantitative real-time PCR |
| *Parkin* | Human | GTGTTTGTCAGGTTCAACTCCA | GAAAATCACACGCAACTGGTC | Quantitative real-time PCR |
| *LC3B* | Human | ACCCTGAGTCTTCTCTTCAGGT | GTTGCGCTTCACAACTCAGG | Quantitative real-time PCR |
| *p62* | Human | GCCATTGCGGAGCCTCATCT | CAGCCATCGCAGATCACATTG | Quantitative real-time PCR |
| *IL6* | Human | CAAATTCGGTACATCCTC | CTGGCTTGTTCCTCACTA | Quantitative real-time PCR |
| *IL8/CXCL8* | Human | GCATAAAGACATACTCCAAACC | AAAACTTCTCCACAACCCTC | Quantitative real-time PCR |
| *MST1* | Human | CCCTGGGAATAACTGCCATA | ATGAAGATTGCCCTCATTGG | Quantitative real-time PCR |
| *MST2* | Human | GCTTGGAGAAGGGTCTTATGG | CATATGGGCTGTCACATTGC | Quantitative real-time PCR |
| *MOB1* | Human | TTCCAGAGGGTTCTCACCAG | CACAGTGTTAACTGCAACCCA | Quantitative real-time PCR |
| *LATS1* | Human | ATACTTGGGGTTGCTGGGAC | ATTAACTCTGGAGGGGAGAGCA | Quantitative real-time PCR |
| *LATS2* | Human | CTCCGCAAAGGGTACACTCA | GAGCGTGTTCTCCCAGTTGA | Quantitative real-time PCR |
| *TAZ* | Human | CAGCCAAATCTCGTGATGAA | TTCTGCTGGCTCAGGGTACT | Quantitative real-time PCR |
| *TEAD1* | Human | GCCCTGGCTATCTATCCACC | TAGACACCTGTTTTCTGGTCCTC | Quantitative real-time PCR |
| *TEAD2* | Human | AGGCTTTCCAGACAATGGCA | AAAAGCTCAGAGGCCTGGAC | Quantitative real-time PCR |
| *TEAD3* | Human | GACCCTGACACGTACAGCAA | GAGCTCCTTCAATCCTCCCT | Quantitative real-time PCR |
| *TEAD4* | Human | GAGCAGAGTTTCCAGGAGG | TCGTTCCGACCATACATCTT | Quantitative real-time PCR |
| *CXCL1* | Human | ACTGCTGCTCCTGCTCCT | CGATGATTTTCTTAACTATGGG | Quantitative real-time PCR |
| *CXCL6* | Human | AGAGCTGCGTTGCACTTGTT | GCAGTTTACCAATCGTTTTGGGG | Quantitative real-time PCR |
| *CCL19* | Human | CTGCTGGTTCTCTGGACTTCC | AGGGATGGGTTTCTGGGTCA | Quantitative real-time PCR |
| *GAPDH* | Human | CTCCTGCACCACCAACTGCT | GGGCCATCCACAGTCTTCTG | Quantitative real-time PCR |

**Table S2. Antibodies used in this study**.

| **Antibodies** | **Source** | **Identifier** | **Application** |
| --- | --- | --- | --- |
| p-YAP1 | Cell Signaling Technology | 4911S | Western blot, immunohistochemistry |
| YAP1 | Cell Signaling Technology | 4912S | Western blot |
| YAP1 | Cell Signaling Technology | 10474S | Immunofluorescence, immunohistochemistry |
| PGC1ɑ | Sigma-Aldrich | AB3242 | Western blot |
| TFAM | Proteintech | 22586-1-AP | Western blot |
| TOMM20 | Proteintech | 11802-1-AP | Western blot |
| PINK1 | Novus Biologicals | BC100-494 | Western blot |
| Parkin | Cell Signaling Technology | 4211S | Western blot |
| Parkin | Cell Signaling Technology | 2132S | Western blot |
| LC3B | Cell Signaling Technology | 2775S | Western blot |
| p62 | Cell Signaling Technology | 5114S | Western blot |
| IL6 | ZEN BIO | 500286 | Western blot |
| CXCL1 | Proteintech | 12335-1-AP | Western blot, immunofluorescence |
| p-MST1/2 | Proteintech | 80093-1-RR | Western blot |
| MST1 | Proteintech | 22245-1-AP | Western blot |
| MST2 | Abcam | ab52641 | Western blot |
| p-MOB1 | Cell Signaling Technology | 8699S | Western blot |
| MOB1 | Cell Signaling Technology | 13730S | Western blot |
| p-LATS1/2 | Cell Signaling Technology | 8654S | Western blot |
| LATS1 | Cell Signaling Technology | 3477T | Western blot |
| LATS2 | Proteintech | 20276-1-AP | Western blot |
| TAZ | Proteintech | 23306-1-AP | Western blot |
| Pan-TEAD | Cell Signaling Technology | 13295S | Western blot |
| OXPHOS antibody cocktail | Proteintech | PK30006 | Western blot |
| β-actin | Cell Signaling Technology | 5125S | Western blot |
| Histone H3 | Proteintech | 17168-1-AP | Western blot |
| GAPDH | Share-Bio Biotechnology | SB-AB2000 | Western blot |
| Anti-rabbit IgG, HRP-linked antibody | Cell Signaling Technology | 7074S | Western blot |
| Anti-mouse IgG, HRP-linked antibody | Cell Signaling Technology | 7076S | Western blot |
| α-SMA | Abcam | ab7817 | Immunohistochemistry |
| KIM1 | Proteintech | 30948-1-AP | Immunohistochemistry |
| CD68 | Servicebio | GB113109 | Immunofluorescence |
| CD206 | Cell Signaling Technology | 24595S | Immunofluorescence |
| Goat anti-Rabbit IgG, secondary antibody, Alexa Fluor 488 | Thermo Fisher | A-11008 | Immunofluorescence |
| Donkey anti-Rabbit IgG, secondary antibody, Alexa Fluor 488 | Thermo Fisher | A-21206 | Immunofluorescence |
| Goat anti-Rabbit IgG H&L (HRP) | Abcam | ab205718 | Immunofluorescence |

**Table S3. Profiling microarray datasets of Gene Expression Omnibus (GEO) used in this study.**

| **GEO ID** | **Species** | **Sample Source** | **Overall Design** | **Groups Definition** | **Platform** | **Year** | **Country** | **Reference** |
| --- | --- | --- | --- | --- | --- | --- | --- | --- |
| GSE104954 | Human | Tubulointerstitial compartment | Tubulointerstitial transcriptome from subjects with chronic kidney disease and living donor biopsies by transcriptome sequencing | (1) Healthy living donor (n=21, GSM2811026-GSM2811028, GSM2811043-GSM2811060)  (2) Diabetic kidney disease (n=17, GSM2810894-GSM2810903, GSM2811029-GSM2811035) | (1) GPL22945 [HG-U133_Plus_2] Affymetrix Human Genome U133 Plus 2.0 Array [CDF: Brainarray HGU133Plus2_Hs_ENTREZG_v19]  (2) GPL22945 [HG-U133A] Affymetrix Human Genome U133A Array (HGU133A Hs ENTREZG 19.0.0) | 2017 | USA | ^12^ |
| GSE30529 | Human | Tubulointerstitial compartment | Tubulointerstitial transcriptome from subjects with chronic kidney disease and living donor biopsies by transcriptome sequencing | (1) Healthy living donor (n=12, GSM757024-GSM757035)  (2) Diabetic kidney disease (n=10, GSM757014-GSM757023) | GPL571 [HG-U133A_2] Affymetrix Human Genome U133A 2.0 Array | 2011 | USA | ^10^ |
| GSE175759 | Human | Tubulointerstitial compartment | Tubulointerstitial transcriptome from subjects with chronic kidney disease and living donor biopsies by transcriptome sequencing | (1) Healthy living donor (n=22, GSM5345948-GSM5345969)  (2) Diabetic kidney disease (n=3, GSM5345970-GSM5345972) | GPL16791 Illumina HiSeq 2500 (Homo sapiens) | 2021 | South Korea | ^11^ |

**Table S4. Profiling single nucleus RNA sequencing datasets of Gene Expression Omnibus (GEO) used in this study.**

| **GEO ID** | **Species** | **Sample Source** | **Overall Design** | **Groups Definition** | **Platform** | **Year** | **Country** | **Reference** |
| --- | --- | --- | --- | --- | --- | --- | --- | --- |
| GSE195460 | Human | Kidney cortex | Single nucleus RNA sequencing (snRNA-seq) of kidney cortex from patients with or without type 2 diabetic kidney disease | (1) Healthy living donor (n=1, GSM5837792)  (2) Diabetic kidney disease (n=2, GSM5837797, GSM5837799) | GPL24676 Illumina NovaSeq 6000 (Homo sapiens) | 2022 | USA | ^15^ |
| GSE151302 | Human | Kidney cortex | Single nucleus RNA sequencing (snRNA-seq) of kidney cortex from patients with or without type 2 diabetic kidney disease | (1) Healthy living donor (n=5, GSM4572192-GSM4572196) | GPL24676 Illumina NovaSeq 6000 (Homo sapiens) | 2020 | USA | ^16, 17^ |
| GSE131882 | Human | Kidney cortex | Single nucleus RNA sequencing (snRNA-seq) of kidney cortex from patients with or without type 2 diabetic kidney disease | (1) Healthy living donor (n=3, GSM3823939-GSM3823941)  (2) Diabetic kidney disease (n=3, GSM3823942-GSM3823944) | GPL24676 Illumina NovaSeq 6000 (Homo sapiens) | 2019 | USA | ^17, 18^ |

**Table S5. Overlapped differentially expressed genes (DEGs) among 3 tubular Gene Expression Omnibus (GEO) datasets.**

| **Overlapped DEGs** | **Genes Names** |
| --- | --- |
| **Upregulated in DKD kidney** | ACKR1/AGR2/ALOX5/ANXA1/ANXA2/APOBEC3B/ARHGAP25/C1QA/C1QB/C1R/C1S/CASP1/**CCL19**/CCND2/CCR2/CD1C/CD1D/CD2/CD209/CD3D/CD48/CD52/CD53/CD74/CLEC10A/CLEC4A/CLU/COMP/CORO1A/CTSS/CX3CR1/**CXCL1**/**CXCL6**/DOCK2/FCER1G/FCGR2B/FCMR/GMFG/GPR18/GZMK/HCK/HCP5/HPGDS/IDO1/IFITM3/ISG20/ITGB2/ITM2C/KLRB1/KRT19/LAPTM5/LCK/LST1/LTB/LY86/MOXD1/MS4A6A/NCF2/P2RY13/PLAC8/PLEKHO1/PLP2/PLTP/PSMB9/PYCARD/RAC2/RARRES1/RASSF2/RFTN1/RRM2/SELL/SLC4A1/TAC1/TGFBI/TIMP1/TMPRSS4/TMSB10/TNFRSF17/TUBA1A/TYROBP//VAV1/VSIG4/VWF/WFDC2 |
| **Downregulated in DKD kidney** | APOLD1/CLDN8/FOSB/HSPB6/KLF9/METAP2/PTGER3/USP2 |

**Table S6. Gene Ontology (GO) analysis of overlapped differentially expressed genes (DEGs) among 3 tubular Gene Expression Omnibus (GEO) datasets.**

| **Description** | **Gene Ratio** | **p.adjust** | **gene ID** |
| --- | --- | --- | --- |
| T cell activation | 24/88 | 1.9964E-15 | ADA/ANXA1/RUNX3/CD1C/CD1D/CD2/CD3D/CD28/CD74/DOCK2/FCER1G/GPR18/NCKAP1L/IDO1/LCK/RAC2/CCL19/VAV1/CORO1A/VSIG4/PYCARD/CD209/CLEC4A/CCR2 |
| leukocyte migration | 22/88 | 7.46694E-13 | ADA/ANXA1/CD2/CD48/CD74/CX3CR1/DUSP1/FCER1G/GPR18/**CXCL1**/NCKAP1L/ITGAM/ITGB2/LCK/RAC2/**CCL19**/**CXCL6**/VAV1/CORO1A/PYCARD/SLAMF8/CCR2 |
| leukocyte cell-cell adhesion | 19/88 | 7.46694E-13 | ADA/ANXA1/RUNX3/CD1D/CD28/CD74/CX3CR1/NCKAP1L/IDO1/ITGB2/LCK/RAC2/CCL19/VAV1/CORO1A/VSIG4/PYCARD/CD209/CCR2 |
| positive regulation of cell activation | 20/88 | 7.46694E-13 | ADA/ANXA1/RUNX3/CD1D/CD2/CD28/CD74/FCER1G/NCKAP1L/ITGAM/ITGB2/LCK/CCL19/VAV1/IRS2/CORO1A/PYCARD/CD209/LILRA5/CCR2 |
| regulation of lymphocyte proliferation | 16/88 | 7.46694E-13 | ADA/ANXA1/CD1D/CD28/CD74/NCKAP1L/IDO1/RAC2/CCL19/LST1/IRS2/CORO1A/VSIG4/PYCARD/CD209/CCR2 |

**Table S7. Kyoto Encyclopedia of Genes and Genomes (KEGG) analysis of overlapped differentially expressed genes (DEGs) among 3 tubular Gene Expression Omnibus (GEO) datasets.**

| **Description** | **Gene Ratio** | **p.adjust** | **gene ID** |
| --- | --- | --- | --- |
| chemokine signaling pathway | 8/59 | 0.002 | CX3CR1/DOCK2/**CXCL1**/RAC2/**CCL19**/**CXCL6**/VAV1/CCR2 |
| phagosome | 7/59 | 0.003 | COMP/ITGAM/ITGB2/NCF2/TAP1/CORO1A/CD209 |
| complement and coagulation cascades | 7/59 | 0.0002 | C1S/C4BPA/CLU/ITGAM/ITGB2/VWF/VSIG4 |
| pertussis | 7/59 | 0.0002 | C1S/C4BPA/CASP1/ITGAM/ITGB2/CXCL6/PYCARD |
| legionellosis | 5/59 | 0.002 | CASP1/CXCL1/ITGAM/ITGB2/PYCARD |


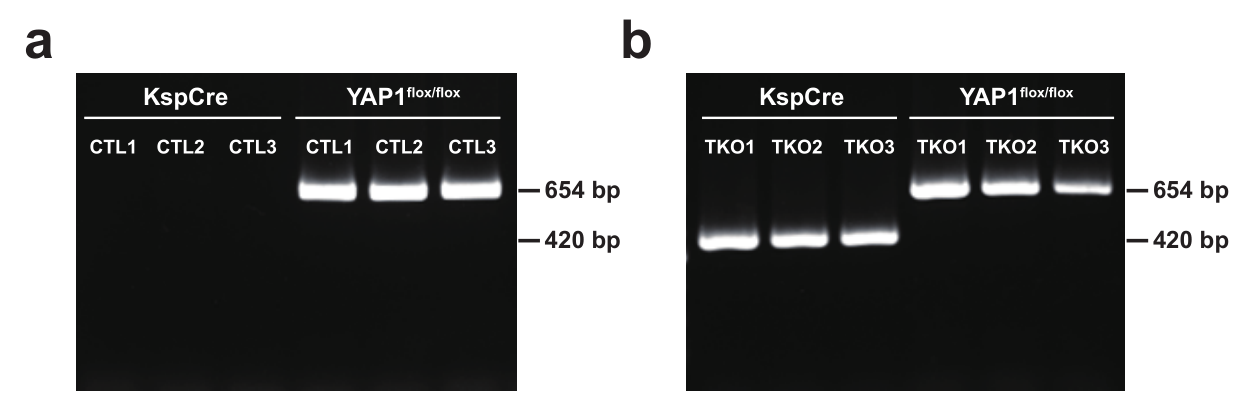


**Fig. S1. Genotyping results of *Yap1* knockout mice (TKO, *KspCre*/*Yap1^flox/flox^*) and their control counterparts lacking the *KspCre* allele (CTL, *WT/Yap1^flox/flox^*). (a)**. Homozygous CTL mice lacking the *KspCre* allele yield only a 654 bp mutant band. (**b)**. Homozygous TKO mice with the *KspCre* allele yield a 654 bp mutant band and a 420 bp Cre band.


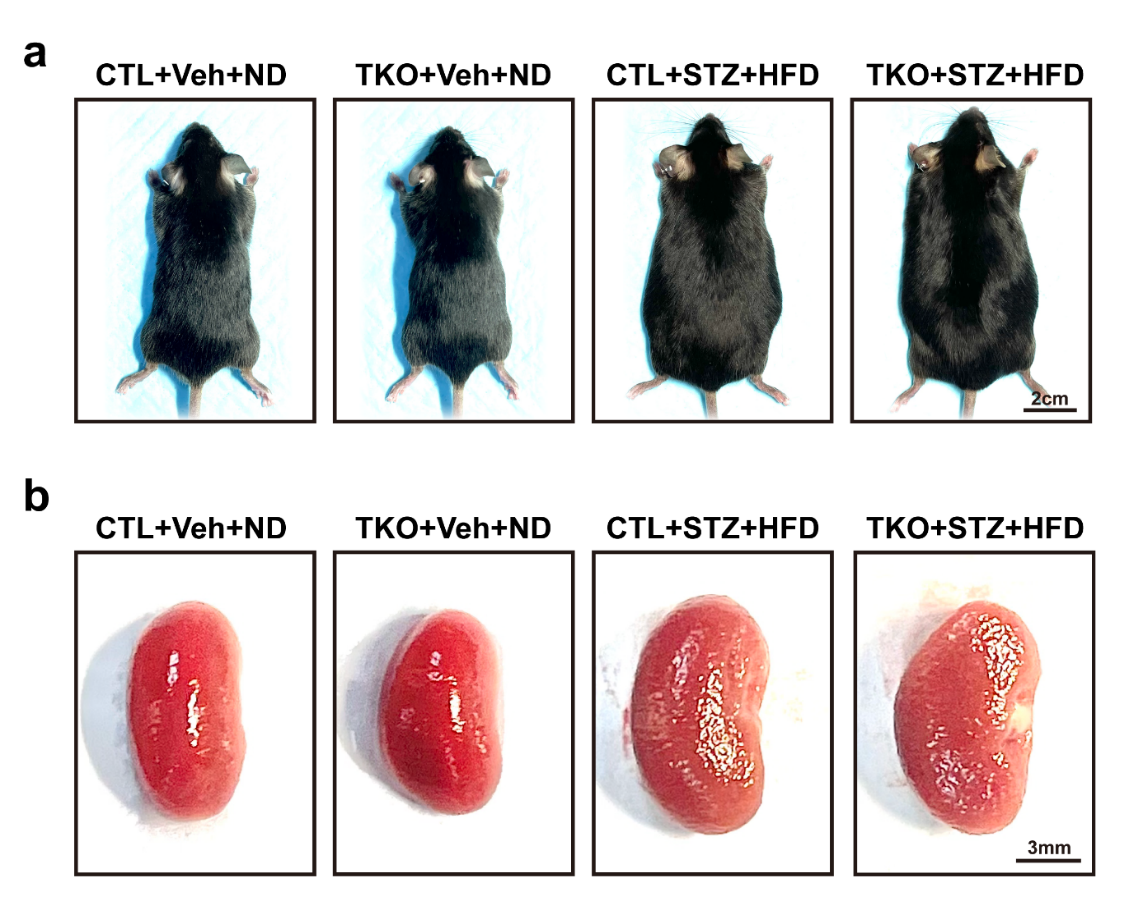


**Fig. S2. Physical appearance and kidney size or morphology of tubule cell-specific *Yap1* knockout mice (TKO, *KspCre*/*Yap1^flox/flox^*) and their control counterparts lacking the *KspCre* allele (CTL, *WT/Yap1^flox/flox^*). (a)**. TKO mice and their CTL littermates displayed normal physical appearance. Diabetic mice induced by STZ+HFD were larger in size as compared to Veh+ND mice. Bar=2cm. (**b)**. There were no discernible differences in kidney size or morphology between TKO mice and their CTL littermates. Kidneys from diabetic mice induced by STZ+HFD were larger in size as compared to Veh+ND group. Bar=3mm. ND, normal diet; HFD, high-fat diet; STZ, streptozotocin; Veh, vehicle.


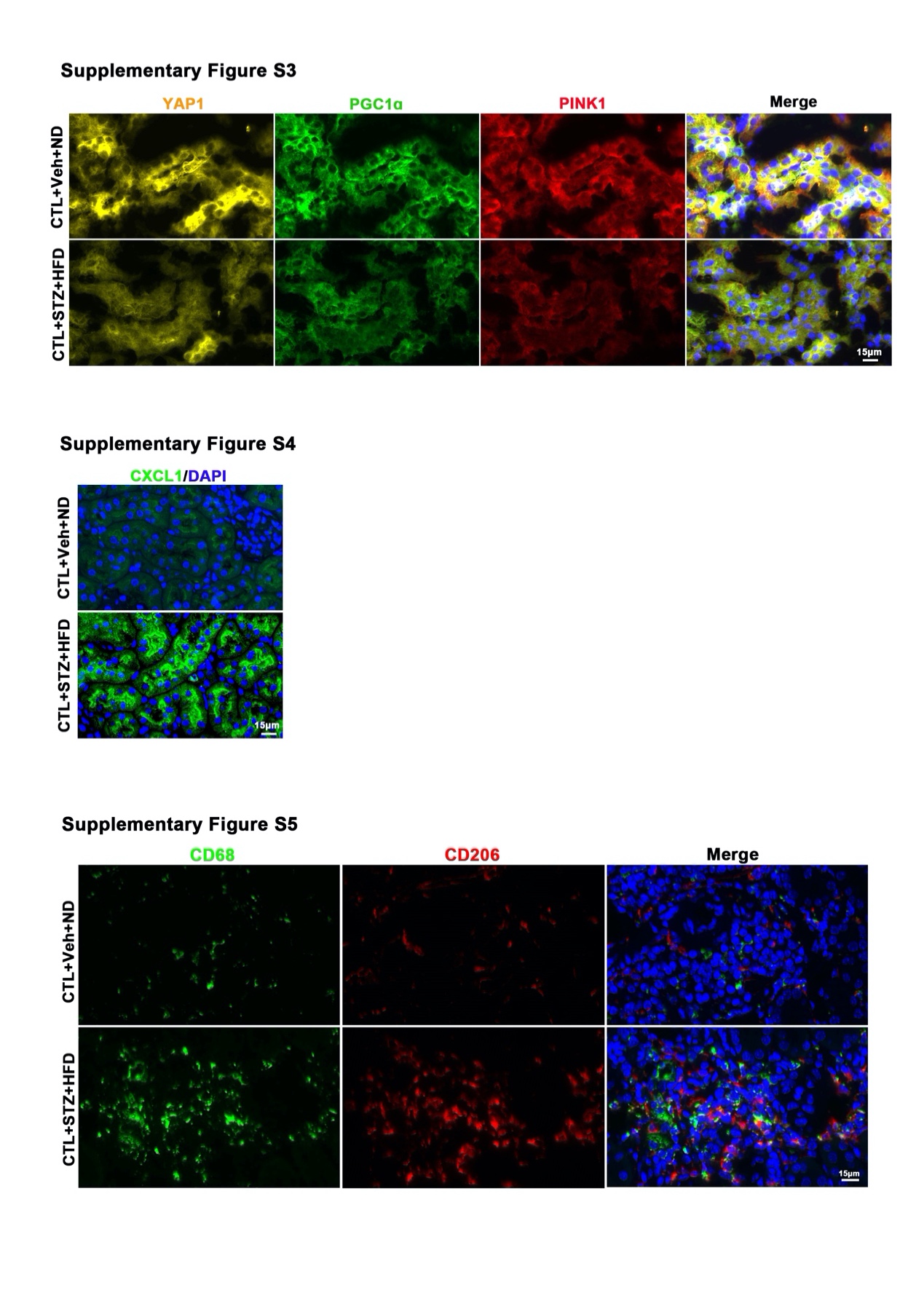


**Fig. S3. CXCL1 secretion was enhanced in tubule cells from mice with diabetic kidney disease (DKD).** Immunofluorescence staining for C-X-C motif chemokine ligand 1 (CXCL1) in kidney sections from indicated mouse groups. Bar=15μm.


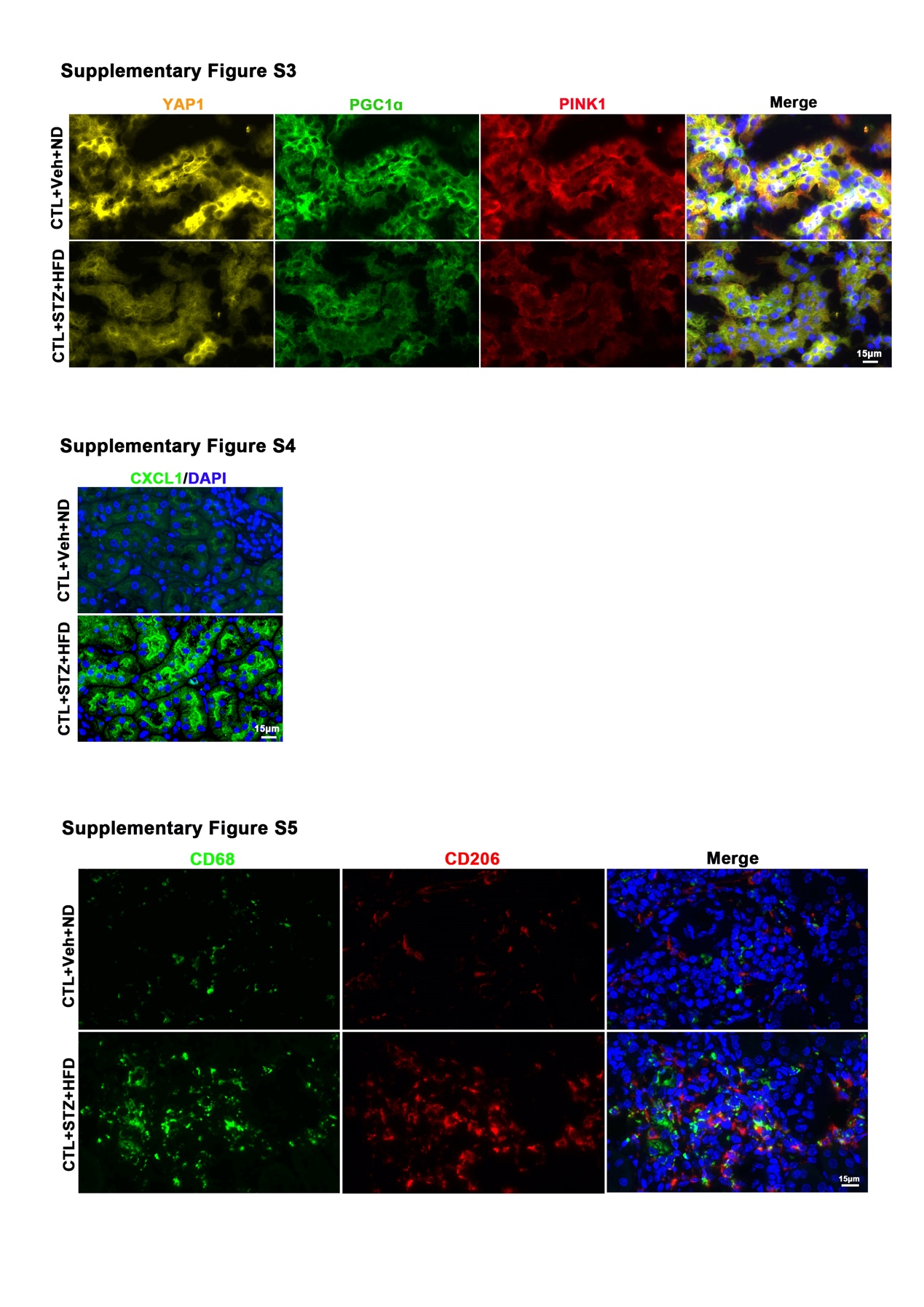


**Fig. S4. Macrophages polarization within kidney microenvironment was heightened in diabetic kidney.** Immunofluorescence staining for CD68 and CD206 in kidney sections from indicated mouse groups. Bar=15μm.

**
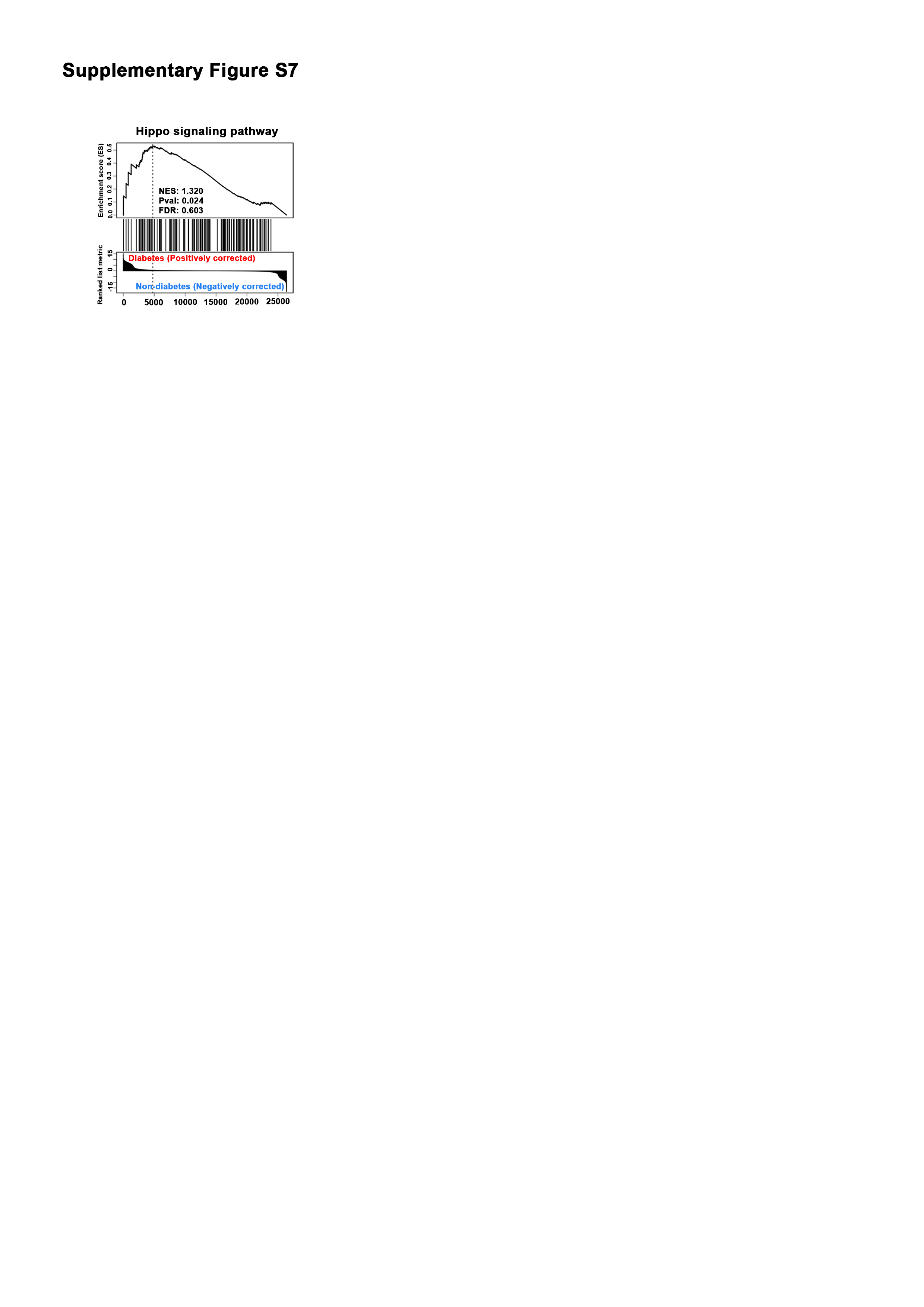
**

**Fig. S5. Gene set enrichment analysis (GSEA) on RNA-sequencing (RNA-seq) data from diabetic mice kidney**. Enrichment plot shows that activation of Hippo signaling pathway was positively associated with diabetes. NES, normalized enrichment score; FDR, false discovery rate.

**
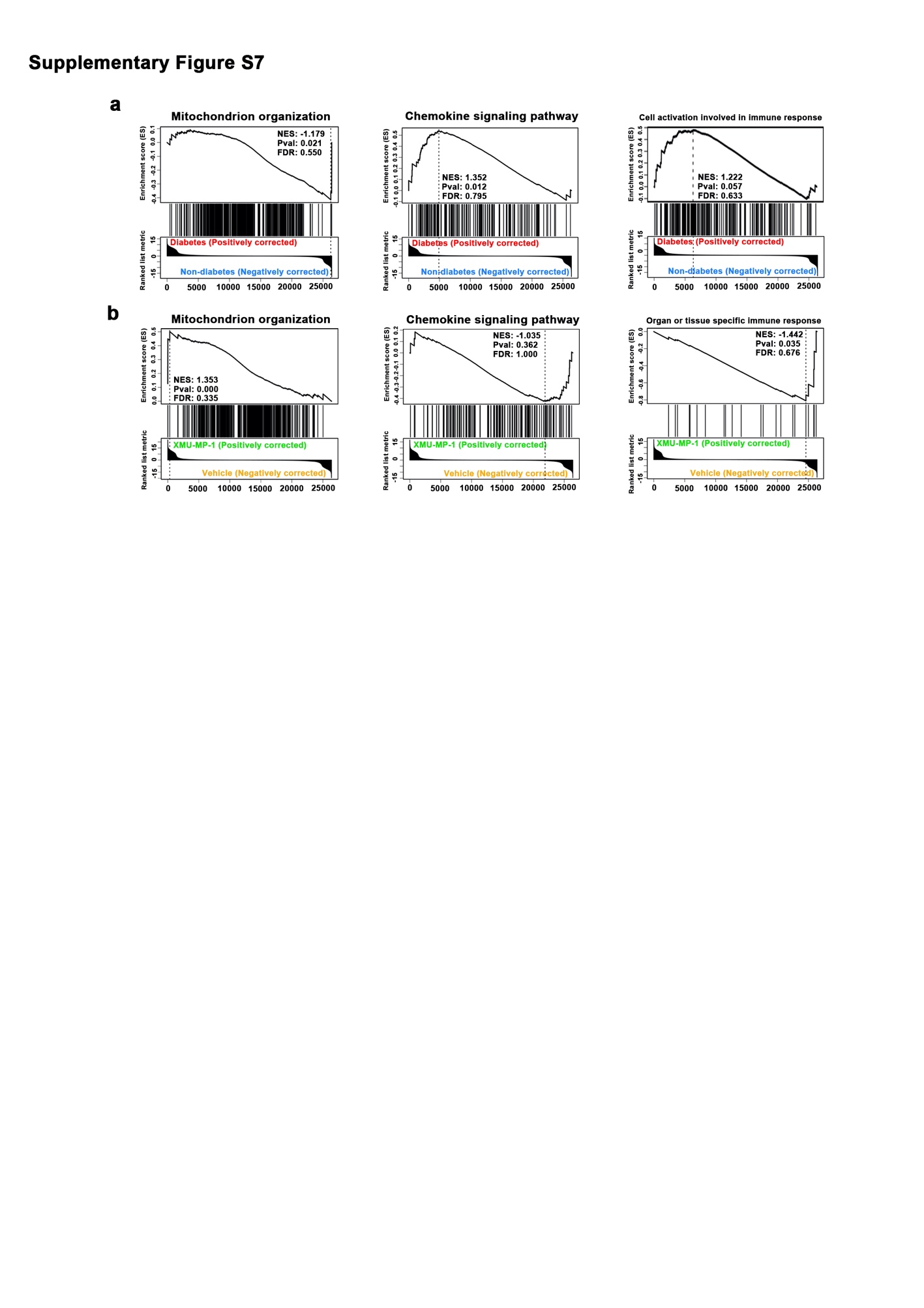
**

**Fig. S6. Gene set enrichment analysis (GSEA) on RNA-sequencing (RNA-seq) data from diabetic mice kidney**. (**a, b**) Enrichment plots summarize some of the major biological and cellular processes including mitochondrion organization, cell activation involved in immune response, and organs or tissue specific immune response, as well as signaling pathway such as chemokine signaling pathway, within tubulointerstitial that are affected by diabetes and XMU-MP-1 administration. NES, normalized enrichment score; FDR, false discovery rate.

**
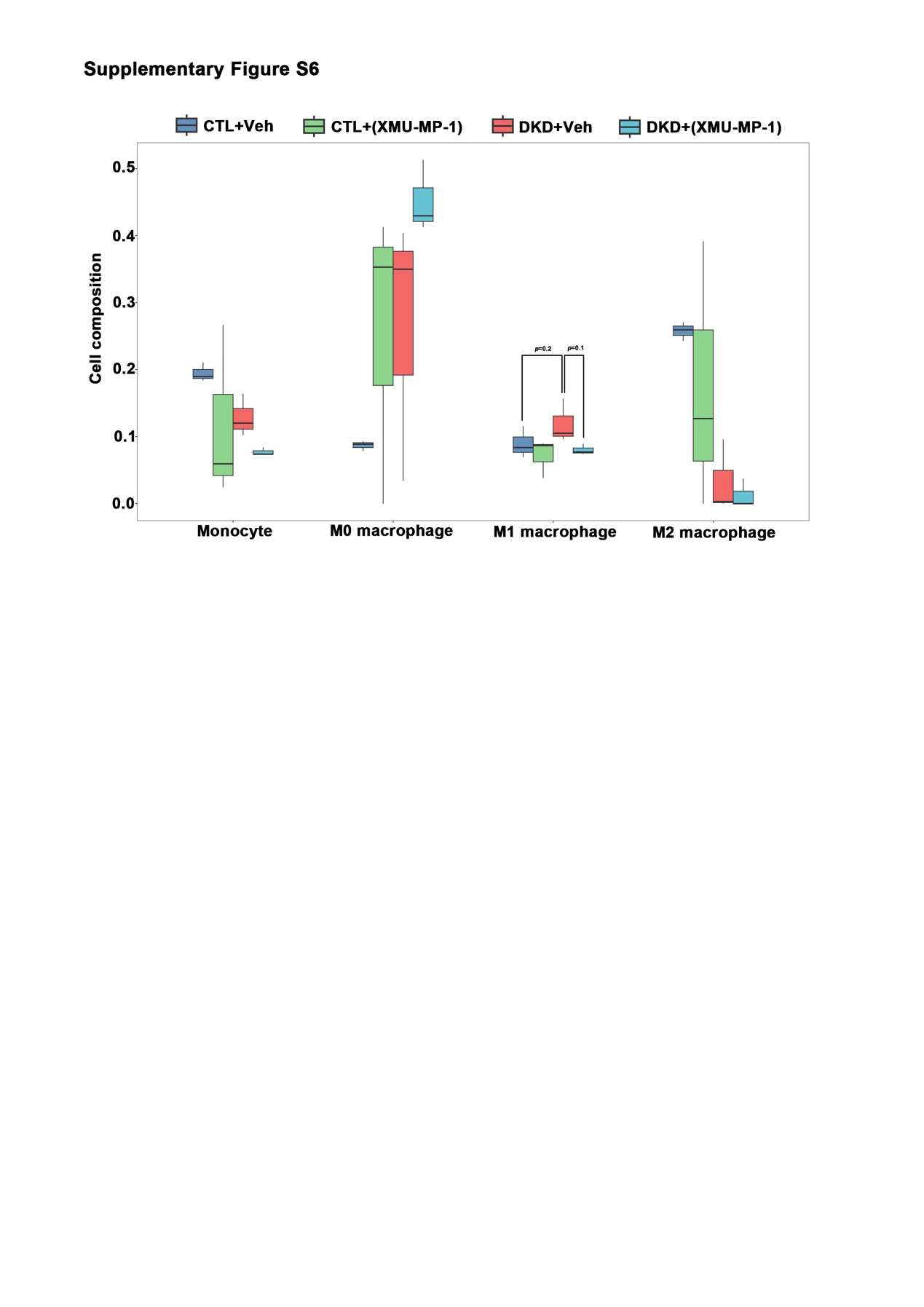
**

**Fig. S7. Composition of infiltrating monocyte and macrophage subpopulation in mice kidney of different groups.** CIBERSORT analysis of RNA-sequencing data of diabetic mice kidney displayed the decreased proportion of M1 macrophage infiltration within kidney microenvironment via administration of XMU-MP-1.


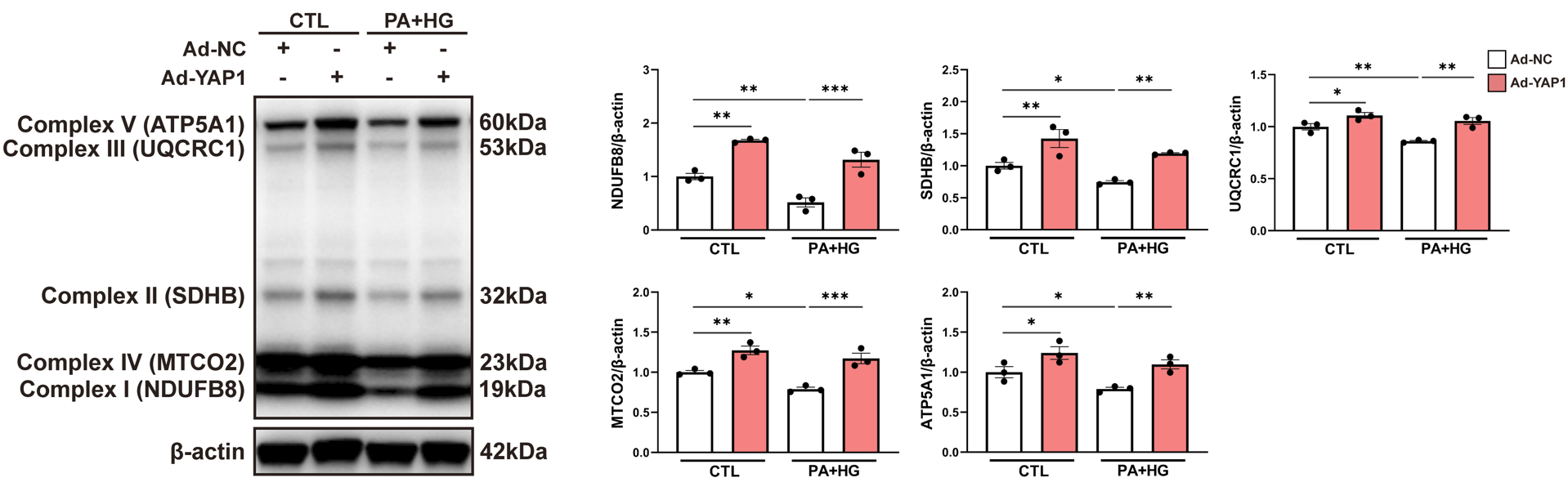


**Fig. S8. Yes-associated protein 1 (YAP1) maintained the expression of subunits of mitochondrial complexes I-V in cultured HK-2 cells**. Representative Western blots and quantification of NDUFB8, SDHB, UQCRC1, MTCO2, and ATP5A1 in adenovirus (Ad)-infected HK-2 cells with or without palmitic acid and high glucose (PA+HG) treatment. Data are expressed as the mean ± SEM. *P < 0.05; **P < 0.01; ***P < 0.001. NC, negative control; NDUFB8, NADH:ubiquinone oxidoreductase subunit B8; SDHB, succinate dehydrogenase complex iron sulfur subunit B; UQCRC1, ubiquinol-cytochrome c reductase core protein 1; MTCO2, mitochondrially encoded cytochrome c oxidase Ⅱ; ATP5A1, ATP synthase F1 subunit alpha.

**References**

1. Lecamwasam A, Novakovic B, Meyer B*, et al.* DNA methylation profiling identifies epigenetic differences between early versus late stages of diabetic chronic kidney disease. *Nephrol Dial Transplant* 2021; 36: 2027-2038.

2. Triastuti E, Nugroho AB, Zi M*, et al.* Pharmacological inhibition of Hippo pathway, with the novel kinase inhibitor XMU-MP-1, protects the heart against adverse effects during pressure overload. *British journal of pharmacology* 2019; 176: 3956-3971.

3. Fan F, He Z, Kong LL*, et al.* Pharmacological targeting of kinases MST1 and MST2 augments tissue repair and regeneration. *Sci Transl Med* 2016; 8: 352ra108.

4. Liu Z, Li Y, Li W*, et al.* Multifunctional Nanohybrid Based on Porous Silicon Nanoparticles, Gold Nanoparticles, and Acetalated Dextran for Liver Regeneration and Acute Liver Failure Theranostics. *Adv Mater* 2018; 30: e1703393.

5. Yue Y, Yeh JN, Chiang JY*, et al.* Intrarenal arterial administration of human umbilical cord-derived mesenchymal stem cells effectively preserved the residual renal function of diabetic kidney disease in rat. *Stem Cell Res Ther* 2022; 13: 186.

6. Palomer X, Pizarro-Delgado J, Barroso E*, et al.* Palmitic and Oleic Acid: The Yin and Yang of Fatty Acids in Type 2 Diabetes Mellitus. *Trends Endocrinol Metab* 2018; 29: 178-190.

7. Helguera P, Seiglie J, Rodriguez J*, et al.* Adaptive downregulation of mitochondrial function in down syndrome. *Cell metabolism* 2013; 17: 132-140.

8. Heo MJ, Suh JH, Lee SH*, et al.* Aryl hydrocarbon receptor maintains hepatic mitochondrial homeostasis in mice. *Molecular metabolism* 2023; 72: 101717.

9. Song H, Tian X, Liu D*, et al.* CREG1 improves the capacity of the skeletal muscle response to exercise endurance via modulation of mitophagy. *Autophagy* 2021; 17: 4102-4118.

10. Woroniecka KI, Park AS, Mohtat D*, et al.* Transcriptome analysis of human diabetic kidney disease. *Diabetes* 2011; 60: 2354-2369.

11. Park S, Lee H, Lee J*, et al.* RNA-seq profiling of tubulointerstitial tissue reveals a potential therapeutic role of dual anti-phosphatase 1 in glomerulonephritis. *J Cell Mol Med* 2022; 26: 3364-3377.

12. Grayson PC, Eddy S, Taroni JN*, et al.* Metabolic pathways and immunometabolism in rare kidney diseases. *Ann Rheum Dis* 2018; 77: 1226-1233.

13. Fan Y, Yi Z, D'Agati VD*, et al.* Comparison of Kidney Transcriptomic Profiles of Early and Advanced Diabetic Nephropathy Reveals Potential New Mechanisms for Disease Progression. *Diabetes* 2019; 68: 2301-2314.

14. Robinson MD, McCarthy DJ, Smyth GK. edgeR: a Bioconductor package for differential expression analysis of digital gene expression data. *Bioinformatics* 2010; 26: 139-140.

15. Wilson PC, Muto Y, Wu H*, et al.* Multimodal single cell sequencing implicates chromatin accessibility and genetic background in diabetic kidney disease progression. *Nature communications* 2022; 13: 5253.

16. Muto Y, Dixon EE, Yoshimura Y*, et al.* Defining cellular complexity in human autosomal dominant polycystic kidney disease by multimodal single cell analysis. *Nature communications* 2022; 13: 6497.

17. Muto Y, Wilson PC, Ledru N*, et al.* Single cell transcriptional and chromatin accessibility profiling redefine cellular heterogeneity in the adult human kidney. *Nature communications* 2021; 12: 2190.

18. Wilson PC, Wu H, Kirita Y*, et al.* The single-cell transcriptomic landscape of early human diabetic nephropathy. *Proceedings of the National Academy of Sciences of the United States of America* 2019; 116: 19619-19625.

19. Song Z, Gao P, Zhong X*, et al.* Identification of Five Hub Genes Based on Single-Cell RNA Sequencing Data and Network Pharmacology in Patients With Acute Myocardial Infarction. *Front Public Health* 2022; 10: 894129.

20. Zhou Q, Huang XR, Yu J*, et al.* Long Noncoding RNA Arid2-IR Is a Novel Therapeutic Target for Renal Inflammation. *Mol Ther* 2015; 23: 1034-1043.

21. Trapnell C, Pachter L, Salzberg SL. TopHat: discovering splice junctions with RNA-Seq. *Bioinformatics* 2009; 25: 1105-1111.
